# ﻿The microsnail genera *Clostophis* and *Acinolaemus* (Eupulmonata, Pupilloidea, Hypselostomatidae) from central Thailand, with description of three new species

**DOI:** 10.3897/zookeys.1258.162797

**Published:** 2025-11-03

**Authors:** Piyoros Tongkerd, Teerangkul Janjai, Arthit Pholyotha, Vukašin Gojšina, Somsak Panha, Chirasak Sutcharit

**Affiliations:** 1 Animal Systematics Research Unit, Department of Biology, Faculty of Science, Chulalongkorn University, Phayathai Road, Patumwan, Bangkok 10330, Thailand Chulalongkorn University Bangkok Thailand; 2 Department of Morphology, Systematics and Phylogeny of Animals, University of Belgrade, Faculty of Biology, Studentski trg 16, 11000, Belgrade, Serbia University of Belgrade Belgrade Serbia; 3 Academy of Science, The Royal Society of Thailand, Bangkok 10300, Thailand The Royal Society of Thailand Bangkok Thailand

**Keywords:** Chao Phraya River, DNA barcoding, gastropods, limestone, taxonomy

## Abstract

Hypselostomatid microsnails of the genera *Clostophis* and *Acinolaemus* from limestone hills in central Thailand were studied and three new species are described. *Clostophis rhynchotes* Tongkerd & Panha, **sp. nov.** is diagnosed by a conical spire, long and descending tuba, 14 spiral striations on the last whorl, and only a single parietal lamella. In addition, a previously known species, *C. proboscideus*, is redescribed, and variations in its apertural dentition are also documented. In the genus *Acinolaemus*, two new sympatric species that clearly differ in shell shape are described. *Acinolaemus rhamphodontis* Tongkerd & Panha, **sp. nov.** is characterised by a depressed conical spire with a long and descending tuba, and eight apertural dentitions, while *A. corusticorus* Tongkerd & Panha, **sp. nov.** possesses a conical shell without a tuba and nine apertural dentitions. Specimens from the type locality of *A. ptychochilus* (the type species), *A. cryptidentatus* and *A. mueangonensis* are re-described and compared with the new species. The living snails of *A. mueangonensis* and *A. rhamphodontis* Tongkerd & Panha, **sp. nov.** possess blackish to translucent bodies. In addition, COI barcoding data for *Clostophis* and *Acinolaemus* are provided for the first time.

## ﻿Introduction

The terrestrial microsnails of Thailand belonging to the family Hypselostomatidae Zilch, 1959 (usually smaller than 5 mm) have been studied for more than 20 years (Panha 1998a, b; Panha and Burch 2005). Currently, this snail group comprises 13 nominal genera, and the species richness in each genus varies from nearly 100 species in *Hypselostoma* Benson, 1856 to one species in *Boysia* Pfeiffer, 1849 and two species in *Pseudostreptaxis* von Möllendorff, 1890 (Schileyko 1998; Páll-Gergely et al. 2019; Jirapatrasilp et al. 2024; Gojšina et al. 2025; MolluscaBase 2025). In the present paper, we focus on the genera *Clostophis* Benson, 1860 and *Acinolaemus* Thompson & Upatham, 1997, which are considered to be closely related and comprise morphologically similar species characterised by small (usually < 2 mm), colourless shells, with or without a detached last whorl (or tuba), and relatively variable apertural dentition (Thompson and Upatham 1997; Páll-Gergely et al. 2020; Páll-Gergely and Hunyadi 2022).

The genus *Acinolaemus* was originally created for eight species and was diagnosed by its colourless shell, its enlarged angular lamella forming a distinctly separated sinulus, and its spiral striation crossed by oblique radial growth lines on the protoconch (Thompson and Upatham 1997; Schileyko 1998). It is believed to be endemic to limestone hills from northern to peninsular Thailand as well as adjactent regions of Myanmar and Malaysia. Subsequently, several species have been added to this genus, so that its distribution range was expanded to include the limestone hills in the Salween Basin in Myanmar, and the Mekong Delta Karsts in Cambodia and Vietnam (Vermeulen et al. 2007, 2019; Changlom et al. 2019; Tongkerd et al. 2024). So far, seven of the 11 currently recognised species of *Acinolaemus* have been recorded in Thailand (Thompson and Upatham 1997; Changlom et al. 2019; MolluscaBase 2025).

The genus *Clostophis* was originally described as a monotypic genus (Benson 1860) that, for more than 150 years, was assigned to the caenogastropod family Diplommatinidae Pfeiffer, 1856, but since the work of Páll-Gergely et al. (2020) and Páll-Gergely and Hunyadi (2022) is now firmly classified as a speciose genus in the heterobranch family Hypselostomatidae. Currently, the genus comprises 20 nominal species which are mainly distributed in the Indo-Burma region, Peninsular Malaysia, and southern China, while only two species have been recorded in Thailand (Páll-Gergely et al. 2020; Páll-Gergely and Hunyadi 2022). *Clostophis proboscideus* (Panha & Burch, 2002) has a precise type locality in central Thailand (Panha and Burch 2002, 2005) while *C. laidlawi* (Collinge, 1902) is only known from the type locality as ‘Jalor’ (= Yala Province, Thailand), although several populations were reported from Malaysia (Collinge 1902; Páll-Gergely and Hunyadi 2022).

Chao Phraya River Basin in central Thailand plays a crucial role in the hydrology and economy of the country. The area is characterised by floodplains, highlands, and scattered limestone hills or karsts (Prasongtham and Kanjanapayont 2014; Singtuen and Phajuy 2020). Limestone hills are unique ecosystems that support significant, highly diverse, and unique biota (Clements et al. 2006). For this study, we surveyed the central Thailand limestone karsts for *Clostophis* and *Acinolaemus* microsnails and collected numerous specimens from multiple localities. Because this material clearly differed in shell shape, shell sculpture, and apertural dentition from all other known *Clostophis* and *Acinolaemus* species, we describe one new *Clostophis* and two new *Acinolaemus* species by means of shell morphology and DNA sequence data. In addition, to contribute to a better understanding of their taxonomy, *C. proboscideus*, *A. ptychochilus* Thompson & Upatham, 1997 (type species of *Acinolaemus*), *A. cryptidentatus*Changlom et al., 2019, and *A. mueangonensis*Changlom et al., 2019 are re-described based on the specimens collected from their type localities.

## ﻿Materials and methods

### ﻿Specimen sampling

The fieldwork was focused on limestone outcrops in the Chao Phraya River Basin in central Thailand. The topography in central Thailand consists of scattered small hills or karst towers above alluvial plains. The flat plains in this region are currently impacted by paddy fields and other agricultural uses, and quarrying.

Specimens were collected from limestone walls, soil, and litter debris at the base of limestone outcrops, and in rock crevices. Shells were soaked in water with detergent, and dirt or mud was removed manually using fine paintbrushes. Dry shells were examined and imaged with a Leica M205C microscope with a fusion optics and the Leica Application Suite Image System. Additional specimens were photographed by scanning electron microscopy (SEM; JEOL, JSM-6610 LV) for more detailed views of microscopic shell structures. Shell whorls were counted to the nearest quarter whorl (Kerney and Cameron 1979). Shell measurements were taken from digital images by Cell’D Imaging Software (Olympus). Identification of specimens and terminology used to describe the apertural dentition were based on Pilsbry (1918, 1948), Páll-Gergely et al. (2020), Páll-Gergely and Hunyadi (2022), Sutcharit et al. (2023), Gojšina et al. (2025), and Sutcharit et al. (2025).

### ﻿DNA extraction, PCR amplification, and sequencing

Total genomic DNA was extracted from tissue samples using the G-spin Genomic DNA Extraction Kit following the manufacturer’s protocol. Approximately 655 bp of the mitochondrial cytochrome c oxidase subunit I (COI) gene was amplified by the polymerase chain reaction (PCR) using the primer pairs LCO1490 and HCO2198 (Folmer et al. 1994).

PCR reactions were performed with an initial denaturation step at 94 °C for 3 min, 35 PCR cycles (94 °C for 45 s, 45–46 °C for 45 s, 72 °C for 1 min) and a final extension step at 72 °C for 5 min. Both strands of the amplified products were purified using the DNA-spin Plasmid DNA Purification Kit and were sequenced at Bioneer Corporation, South Korea. Chromatograms were manually corrected for misreads, if necessary, and forward and reverse strands were merged into one sequence file using MEGA v. 7.0 (Kumar et al. 2016). New sequences have been deposited in GenBank (Table 1). Additionally, twelve COI sequences were downloaded from GenBank (Table 1). Ten sequences from six *Hypselostoma* species were used as part of the ingroup and two sequences from two *Pupilla* species were used as more distantly related outgroup.

**Table 1.** Samples used for phylogenetic analysis, with specimen codes, museum registration numbers, GenBank accession numbers, sampling localities, and references. 1 = Hoekstra and Schilthuizen (2011). 2 = Nekola et al. (2015).

Taxon	Code	Museum registration number	GenBank accession number (COI)	Locality	Reference
*Acinolaemus rhamphodontis* sp. nov.	HC025-1	Paratype CUMZ 14451	PV698334	Tak, Thailand	this study
*Acinolaemus rhamphodontis* sp. nov.	HC025-2	Paratype CUMZ 14451	PV698335	Tak, Thailand	this study
*Acinolaemus mueangonensis* Changlom et al., 2019	HC026	CUMZ 144554	PV698336	Tak, Thailand	this study
*Acinolaemus colpodon* Thompson & Upatham, 1997	HE022-2	CUMZ 14445	PV698337	Chon Buri, Thailand	this study
*Acinolaemus rhamphodon* Thompson & Upatham, 1997	HE025-2	CUMZ 14481	PV698338	Chon Buri, Thailand	this study
*Clostophis rhynchotes* sp. nov.	HC016-2	Paratype CUMZ 14464	PV698339	Nakhon Sawan, Thailand	this study
*Clostophis udayaditinus* Sutcharit & Panha, 2025	PUP020	Paratype CUMZ 14467	PV698340	Battambang, Cambodia	this study
*Hypselostoma hungerfordianum* von Möllendorff, 1891	-	-	HM240390	Kelantan, Malaysia	1
*Hypselostoma hungerfordianum* von Möllendorff, 1891	-	-	HM240394	Perak, Malaysia	1
*Hypselostoma salpinx* (van Benthem Jutting, 1961)	-	-	HM240407	Pahang, Malaysia	1
*Hypselostoma salpinx* (van Benthem Jutting, 1961)	-	-	HM240408	Pahang, Malaysia	1
*Hypselostoma frequens* (van Benthem Jutting, 1950)	-	-	HM240409	Kelantan, Malaysia	1
*Hypselostoma depressispira* (van Benthem Jutting, 1949)	-	-	HM240410	Kelantan, Malaysia	1
*Hypselostoma serpa* (van Benthem Jutting, 1950) (= *Paraboysidia tarutao* Panha & Burch, 2002)	-	-	HM240411	Kedah, Malaysia	1
*Hypselostoma serpa* (van Benthem Jutting, 1950) (= *Paraboysidia tarutao* Panha & Burch, 2002)	-	-	HM240412	Kedah, Malaysia	1
*Hypselostoma transitans* von Möllendorff, 1894	-	-	HM240405	Kedah, Malaysia	1
*Hypselostoma transitans* von Möllendorff, 1894	-	-	HM240406	Kedah, Malaysia	1
*Pupilla loessica* Ložek, 1954	-	-	KM518576	Belyashi, Altai, Russia	2
*Pupilla triplicata* (Studer, 1820)	-	-	KM518612	Hracholusky, Bohemia, Czech Republic	2

### ﻿Sequence alignment and phylogenetic analysis

Sequences were aligned using MEGA v. 7.0 with the default settings (Kumar et al. 2016). COI-sequence divergence was expressed as *p*-distances, calculated with MEGA v. 7.0 with the ‘pairwise deletion of gaps’ option (Kumar et al. 2016). Bayesian inference (BI) and maximum likelihood (ML) analyses were used to reconstruct phylogenetic relationships through the online CIPRES Science Gateway (Miller et al. 2010). The best-fit model of nucleotide substitution was identified for each sequence partition separately by means of the corrected Bayesian information criterion (BIC) using Kakusan4 (Tanabe 2011). As suggested by the Kakusan4 program, three partitions were designated: the HKY85 model with a gamma distribution for the first codon position of the COI fragment, and the GTR model with a gamma distribution for the second and the third codon positions of the COI fragment. Bayesian inference phylogenies were generated using MrBayes on ACCESS v. 3.2.7a (Ronquist et al. 2012), with two runs for 10 million generations, sampling every 1000 generations, and the first 25% of obtained trees being discarded as burn-in. Maximum likelihood phylogenetic trees were generated using RAxML-HPC2 on ACCESS v. 8.2.12 (Stamatakis 2014) using a GTRCAT model. Bootstrap support (BS) values were used for assessing branch support in the ML tree with 1000 bootstrap replicates and shown as a percentage. Branch support in the BI tree was evaluated by posterior probabilities (PP). PP values ≥ 0.95 and BS values ≥ 70% were interpreted as positive support for nodes, while lower values were interpreted as weak support. Both ML and BI phylogenetic trees were visualised and modified in FigTree v. 1.4.4 (Rambaut 2018), then visually processed in Adobe Illustrator 2021.

### ﻿Institutional abbreviations

**CUMZ**Chulalongkorn University, Museum of Zoology, Bangkok;

**NHMUK**The Natural History Museum, London;

**SMF**Senckenberg Forschungsinstitut und Naturmuseum, Frankfurt am Main;

**UF**Florida Museum of Natural History, University of Florida.

### ﻿Abbreviations

**a**, angular lamella; **b**, basal plica; **c**, columellar lamella; **ifpl**, infrapalatal plica; **ip**, infraparietal lamella; **itpl**, inter palatal plica; **lpl**, lower palatal plica; **p**, parietal lamella; **pt**, palatal tubercle; **sbc**, subcolumellar lamella; **spc**, supracolumellar lamella; **upl**, upper palatal plica.

## ﻿Results

### ﻿Molecular phylogenetic analyses

Our final dataset contained COI sequences from 17 specimens representing 12 taxa in the Hypselostomatidae. Seven species were represented by just a single specimen and five species were represented by two specimens. Additionally, two sequences from two pupillid taxa, *Pupilla loessica* Ložek, 1954 and *P. triplicata* (Studer, 1820), were included as outgroup. The final COI alignment had a total length of 655 bp, containing 264 variable sites, 391 invariant sites, 232 parsimony-informative sites, and 32 singleton sites.

**Figure 1.**
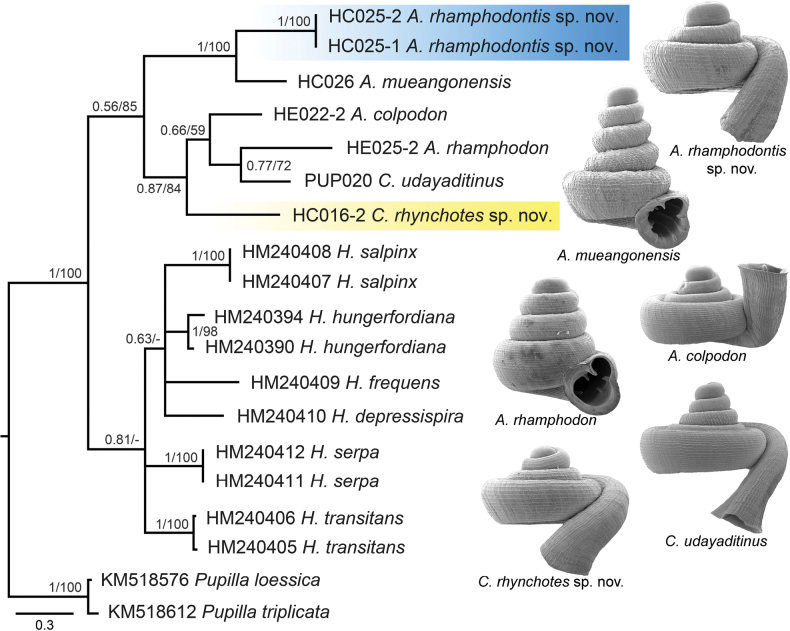
Bayesian inference tree from analysis of the mitochondrial cytochrome c oxidase subunit I (COI) sequence data of species in *Clostophis*, *Acinolaemus*, and *Hypselostoma*. Numbers on branches indicate the Bayesian posterior probabilities (PP) and ML bootstrap (BS) support values. Blue colour represents *Acinolaemus rhamphodontis* sp. nov. clade. Yellow colour represents *Clostophis rhynchotes* sp. nov. clade. Representative shells of *Clostophis* and *Acinolaemus* are shown but not to scale.

The BI and ML trees were largely consistent; therefore, only the BI tree is presented in Fig. 1. Both BI and ML analyses supported the monophyly of the family Hypselostomatidae (BB/PP: 1/100). However, the phylogenetic relationships within this family remain unresolved. Although six species of *Hypselostoma* formed a monophyletic group in the BI analysis with weak nodal support, they were not retrieved as a monophyletic group in the ML tree. Similarly, the monophyly of the genera *Clostophis* and *Acinolaemus* was not supported. Conversely, the sister group relationship of *A. rhamphodontis* sp. nov. and *A. mueangonensis* was well-supported (BB/PP: 1/100). In contrast, the remaining *Acinolaemus* species (*A. rhamphodon* and *A. colpodon*) were grouped together with two *Clostophis* species (*C. rhynchotes* sp. nov. and *C. udayaditinus*) in a separate clade (BB/PP: 0.87/84). This phylogenetic pattern suggests that current generic boundaries between *Clostophis* and *Acinolaemus* may not accurately reflect their evolutionary relationships and highlights the need for further taxonomic revision within the group.

The mean interspecific *p*-distance among the four *Acinolaemus* species varied from 14.4% to 18.4% and no intraspecific sequence divergence within our examined specimens of *A. rhamphodontis* sp. nov. (Table 2). The *p*-distance between *C. rhynchotes* sp. nov. and *C. udayaditinus* was 15.6% (Table 2). The average interspecific *p*-distance for the six *Hypselostoma* species varied from 13.7% to 21.9% and the average intraspecific sequence divergence within *Hypselostoma* varied from 0% to 6.7% (Table 2).

**Table 2.** Intra- and interspecific COI *p*-distances among *Acinolaemus*, *Clostophis*, and *Hypselostoma*.

Taxa	1	2	3	4	5	6	7	8	9	10	11	12
1. *A. rhamphodontis* sp. nov.	0											
2. *A. rhamphodon*	0.184	-										
3. *A. colpodon*	0.167	0.144	-									
4. *A. mueangonensis*	0.155	0.183	0.179	-								
5. *C. rhynchotes* sp. nov.	0.190	0.163	0.134	0.175	-							
6. *C. udayaditinus*	0.179	0.131	0.131	0.184	0.156	-						
7. *H. transitans*	0.210	0.187	0.182	0.196	0.192	0.197	0.031					
8. *H. serpa*	0.174	0.172	0.164	0.190	0.163	0.173	0.137	0.003				
9. *H. salpinx*	0.204	0.192	0.168	0.209	0.185	0.197	0.167	0.147	0			
10. *H. hungerfordianum*	0.182	0.176	0.154	0.179	0.170	0.181	0.139	0.137	0.151	0.067		
11. *H. frequens*	0.219	0.218	0.192	0.212	0.202	0.214	0.179	0.166	0.169	0.147	-	
12. *H. depressispira*	0.198	0.198	0.183	0.195	0.177	0.197	0.161	0.166	0.145	0.139	0.179	-

### ﻿Systematics

﻿**Family Hypselostomatidae Zilch, 1959**

#### Clostophis

Taxon classificationAnimaliaStylommatophoraHypselostomatidae

Benson, 1860

9BD9F1E4-62F7-57E8-ADBF-CA71858C7F37

Clostophis Benson, 1860: 95. Kobelt 1902: 484. Thiele 1929: 111. Páll-Gergely et al. 2020: 351, 352. Páll-Gergely and Hunyadi 2022: 419. Preece et al. 2022: 145, 256.
Montapiculus Panha & Burch, 2002: 148. Type species: Montapiculus proboscidea Panha & Burch, 2002. Panha and Burch 2005: 38, 109.

##### Type species.

*Clostophis sankeyi* Benson, 1860, by monotypy.

##### Remarks.

The genus was recently revised by Páll-Gergely et al. (2020) and Páll-Gergely and Hunyadi (2022), who recorded two species from southeastern Myanmar, six species from Laos, six species from Vietnam, two species from Peninsular Malaysia, and two species from Thailand. All these species share strong spiral ridges, usually with one or many apertural dentitions (except for four species with no dentition), and a more or less detached last whorl (tuba). Other characters, such as shell shape and tuba length, show large intraspecific variation (although they can be useful in some cases to distinguish species). Based on its wide distribution and its substantial morphological variability, the genus can be divided into three phenotypic species groups:

**Table 3.** Species list, diagnostic characters, and type localities of *Clostophis* species. Numbers in species column refer to those in Figure 2A. The superscript numbers indicate references: 1 = Collinge (1902); 2 = van Benthem Jutting (1961); 3 = Panha and Burch (2002); 4 = Páll-Gergely et al. (2015); 5 = Páll-Gergely et al. (2017); 6 = Páll-Gergely et al. (2020); 7 = Páll-Gergely and Hunyadi (2022); 8 = Sutcharit et al. (2025); 9 = this study. n/a = data not available.

Species	Shell shape	Tubular	Protoconch	Aperture opening	Apertural dentition	Type locality
***C. sankeyi* species group**						
1 *C. sankeyi* Benson, 1860^6^	concave-conical	long descending	pitted and spirally striated	ventral to subventral	1 (parietal) or 2 (parietal, palatal)	Myanmar, Mon State
2 *C. proboscideus* (Panha & Burch, 2002)^3,6,9^	concave-conical	long and descending	pitted and spirally striated	ventral to subventral	2 (parietal, palatal)	Thailand, Nakornsawan
3 *C. rhynchotes* sp. nov.^9^	concave-conical	long and descending	pitted and spirally striated	ventral to subventral	1 parietal	Uthai Thani, Thailand
4 *C. udayaditinus* Sutcharit & Panha, 2025^8^	concave-conical	long and descending	pitted and spirally striated	ventral to subventral	4 (parietal, infraparietal, palatal, columellar)	Cambodia, Battambang
5 *C. yoga* Páll-Gergely & Hunyadi, 2022^7^	concave-conical	long and descending	spiral striations	subventral	none	Vietnam, Thanh Hoa
***C. charybdis* species group**						
6 *C. charybdis* Páll-Gergely & Hunyadi, 2022^7^	concave-conical	very short to absent	pitted and spirally striated	lateral	2 (parietal, palatal)	Vietnam, Lang Son
7 *C. candidus* Páll-Gergely & Hunyadi, 2022^7^	conical, slightly concave side	short	spirally striated	sublateral	none	Vietnam, Lang Son
8 *C. incurvus* Páll-Gergely & Vermeulen, 2020^6^	conical, straight to slightly convex side	very short to absent	n/a	lateral	2 (parietal, palatal)	Vietnam, Quang Ninh
9 *C. infantilis* Páll-Gergely, 2020^7^	conical, straight side	absent	pitted and spirally striated	lateral	none	Vietnam, Haiphong
10 *C. koilobasis* Páll-Gergely & Vermeulen, 2020^6^	low conical, concave side	very short to absent	spirally striated	lateral	1 parietal	Laos, Khammouan
11 *C. multiformis* Páll-Gergely & Reischütz, 2020^6^	conical, straight side	short to slightly long	pitted and spirally striated	lateral or sublateral	1 parietal	Laos, Khammouane
12 *C. neglectus* (van Benthem Jutting, 1961)^2,6^	conical, straight side	very short to absent	spirally striated	lateral	2 (parietal, palatal)	Malaysia, Pahang
13 *C. obtusus* Páll-Gergely & Grego, 2020^6^	conical, straight to slightly convex side	very short to absent	pitted and spirally striated	lateral	none	Laos, Khammouane
14 *C. platytrochus* Páll-Gergely & Hunyadi, 2020^6^	conical, slightly concave side	short and descending	spirally striated	sub lateral	2 (parietal, palatal)	Vietnam, Da Nang
15 *C. stochi* (Páll-Gergely & Jochum, 2017)^5^	conical, slightly convex side	very short to absent	spirally striated	lateral	2 (parietal, palatal)	Vietnam, Ha Giang
16 *C. thinbowguensis* Páll-Gergely & Hunyadi, 2022^7^	conical, straight side	short	n/a	sublateral	1 parietal	Myanmar, Tanintharyi
***C. bactrianus* species group**						
17 *C. bactrianus* Páll-Gergely & Hunyadi, 2022^7^	conical, straight side	short	spirally striated	lateral	4 (parietal, palatal, basal, columellar)	Malaysia, Pahang
18 *C. lacrima* (Páll-Gergely & Hunyadi, 2015)^4^	concave-conical	very short to absent	pitted and spirally striated	lateral	4 (parietal, palatal, basal, columellar)	China, Guangxi
19 *C. laidlawi* (Collinge, 1902)^1,7^	conical, slightly concave side	very short to long and descending	spirally striated	lateral or sublateral	5 (parietal, angular, palatal, basal, columellar)	Thailand, Yala
20 *C. obliquus* Páll-Gergely & Hunyadi, 2022^7^	conical, slightly concave side	very short to absent	pitted and spirally striated	lateral	4 (parietal, palatal, basal, columellar)	China, Guangxi
21 *C. socialis* (Páll-Gergely & Hunyadi, 2015)^4^	concave-conical	short	pitted and spirally striated	lateral	4 (parietal, palatal, basal, columellar)	China, Guangxi

**Figure 2.**
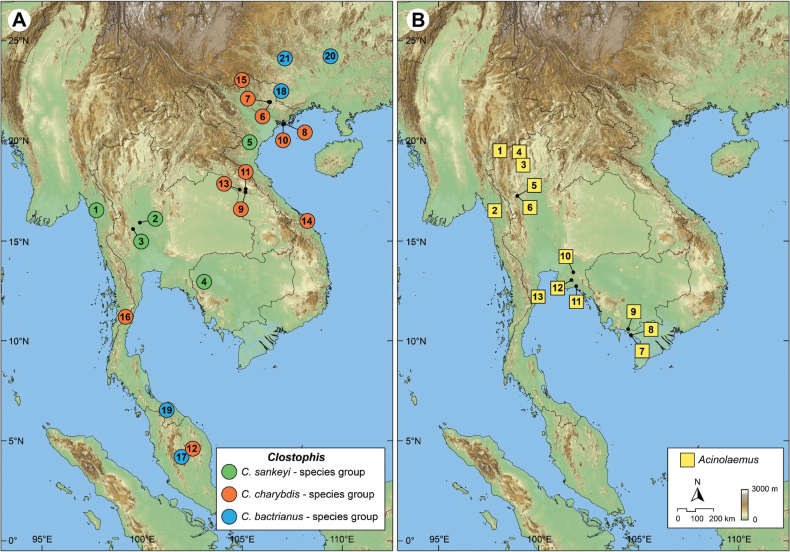
Approximate type localities. A. All *Clostophis* species. Colours indicate different species groups; numbers correspond to the species listed in Table 3; B. All *Acinolaemus* species; numbers correspond to the species listed in Table 4.

1. *Clostophis sankeyi* species group: with 0–4 apertural dentitions, a long (~1/4 whorl or more) and descending tuba, and a conical shell shape with strongly concave sides. This group consists of five species (including *Clostophis rhynchotes* sp. nov.) distributed in Myanmar, Thailand, and northern Vietnam (Fig. 2A, Table 3).
2. *Clostophis charybdis* species group: with 0–2 apertural dentitions, no to short tuba, moderately to strongly conical shell, concave sides, and aperture opened laterally to sublaterally. This species group comprises ten species, of which nine species are distributed mainly in the central to northern Annamite Ranges in Laos and central to northern Vietnam; one species is found in southern Myanmar (Fig. 2A, Table 3).
3. *Clostophis bactrianus* species group: with four or five strong apertural dentitions, absent to short tuba, and conical to slightly concave sides. This group consists of five species with disjunct distributions. Three species from southern China tend to have long denticles deeper inside the aperture, whereas the two species from Peninsular Malaysia tend to have short denticles situated near the apertural lip (Fig. 2A, Table 3).

#### ﻿ Clostophis proboscideus

Taxon classificationAnimaliaStylommatophoraHypselostomatidae

(Panha & Burch, 2002)

523EAF63-BD27-5CC3-823F-0ECAC475A586

**Figure 3.**
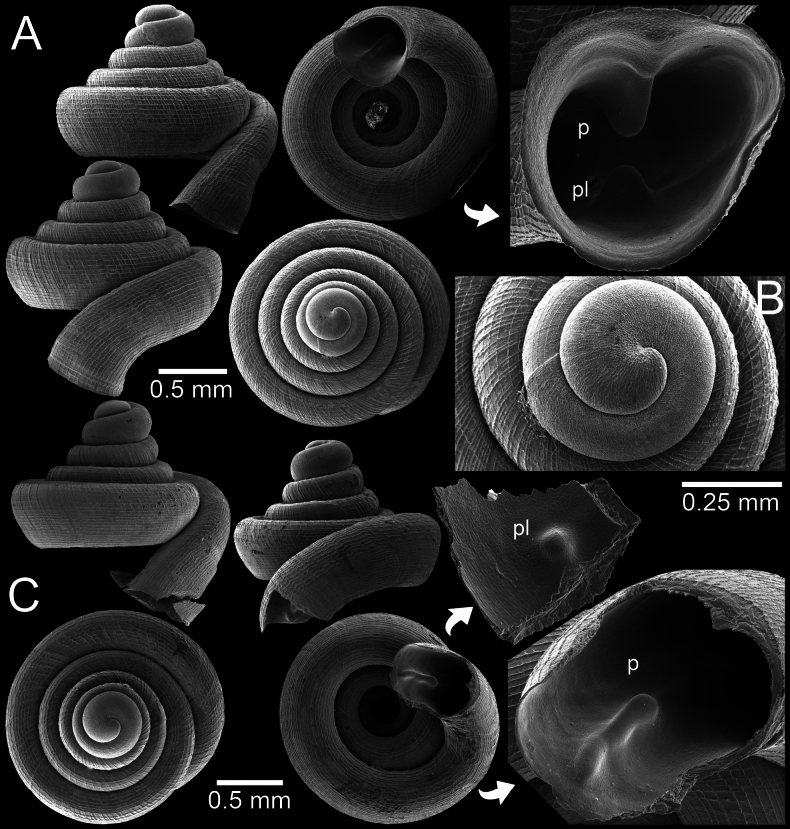
*Clostophis proboscideus*, paratype CUMZ 14457 (former Ver-080) from the type locality. A. Shell from different angles and showing enlarged aperture; B. Protoconch and earlier whorl sculptures; C. Shell from different angles and showing enlarged aperture. Note: the aperture of this shell was broken during the SEM imaging process.

**Figure 4.**
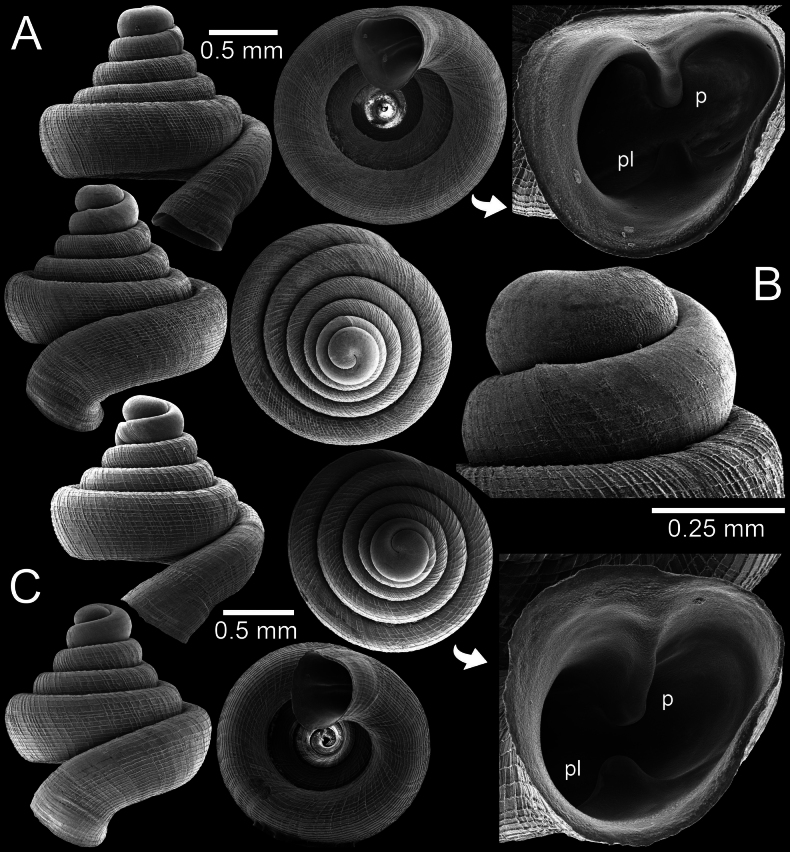
*Clostophis proboscideus*, topotype CUMZ 14456 from the type locality showing variation in shell shape and sculpture. A. Shell with dense spiral striae sculpture; B. Protoconch and earlier whorl sculptures; C. Shell with conical spire with strong spiral striae and radial ridges.

**Figure 5.**
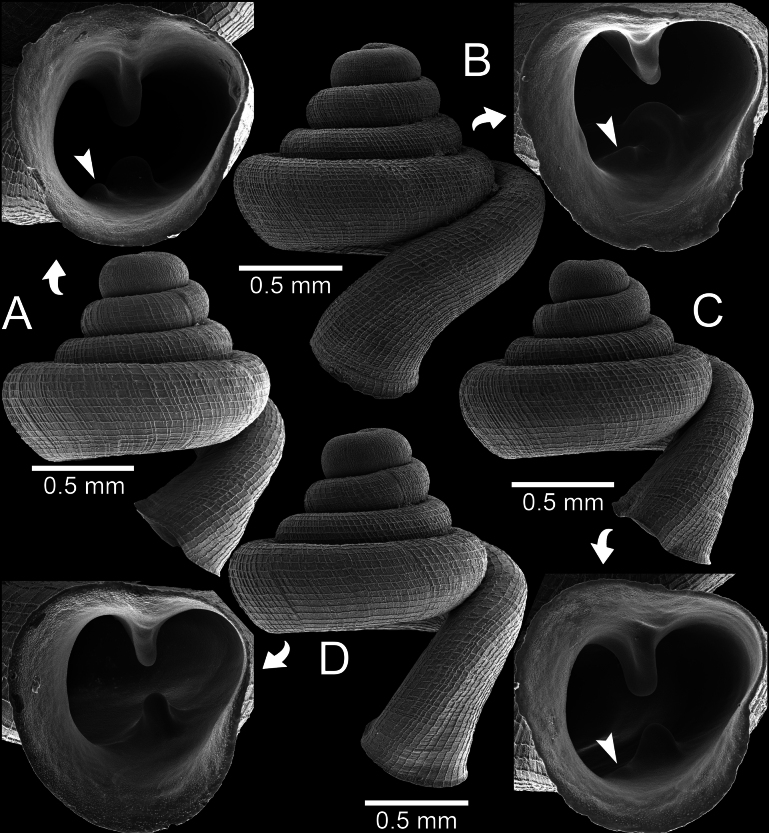
*Clostophis proboscideus*, specimen CUMZ 14429 from Khao Pathawee, Uthai Thani Province, intra population variation in basal lamella. A. Specimen with prominent infrapalatal plica; B. Specimen with low but noticeable infrapalatal plica; C. Specimen with inconspicuous infrapalatal plica; D. Specimens without infrapalatal plica. White arrowhead indicates position of infrapalatal plica.

Figs 3, 4, 5, Table 3

Montapiculus proboscidea Panha & Burch, 2002 [1999]: 148, figs 4–7. Type locality: Teppratan mountain, Nakornsawan Province. Panha and Burch 2005: 109, fig. 94.
Clostophis proboscideus —Páll-Gergely et al. 2020: 364, figs 1j, 3b.

##### Material examined.

***Holotype*.** Thailand • Wat Khao Huai Lung (former Teppratan mountain), Ban Daen, Banphot Phisai District, Nakhon Sawan Province; 15°55'35.4"N, 99°52'23.2"E; S. Panha leg.; CUMZ 14458 (former Ver-079). ***Paratypes*.** Thailand • 2 shells (Fig. 3A–C); same data as for holotype; CUMZ 14457 (former Ver-080).

##### Other material.

Thailand • 2 shells (Fig. 4A–C); Wat Khao Huai Lung (former Teppratan mountain), Ban Daen, Banphot Phisai District, Nakhon Sawan Province; 15°55'35.4"N, 99°52'23.2"E; P. Tongkerd leg.; CUMZ 14456. • 2 shells + 2 juveniles; Wat Tham Bo Ya, Nong Krot, Mueang Nakhon Sawan District, Nakhon Sawan Province; 15°43'49.7"N, 99°56'45.7"E; P. Tongkerd leg.; CUMZ 14465. • 4 shells + 1 juvenile (Fig. 5A–D); Khao Pathawee, Taluk Du, Thap Than District, Uthai Thani Province; 15°28'25.2"N, 99°45'30.3"E; P. Tongkerd leg.; CUMZ 14429. • 105 shells + 8 juveniles; same collection data as preceding; CUMZ 14463. • 4 shells; Phu Toei, Huai Khamin, Dan Chang District, Suphan Buri Province; S. Panha leg.; CUMZ 15351.

##### Diagnosis.

Shell concave-conical and with long and descending tuba. Apertural dentition with one parietal lamella and one weak palatal plica. Umbilicus wide.

##### Description.

Shell concave-conical, colourless; growing regularly; last whorl slightly expanded. Shell height 1.7–1.9 mm (including tuba) and shell width 1.6–1.7 mm. Apex large and rounded; protoconch ~1½ whorls, pitted and with very narrow spiral striae. Whorls ~4½–5 (excluding tuba), rounded and convex; suture wide and deep. Shell surface with strong, elevated and rather equidistant spiral striae (18–22 on body whorl in frontal view), and crossed with weak to strong and irregular radial growth lines. Sometimes growth lines on earlier whorls strong, thus making reticulated surface. Penultimate whorl seemingly sunken into last whorl. Last whorl slightly shouldered to rounded; tuba long, ~¼ whorl, strongly descending, curving and slightly twisted. Aperture subovate, open ventrally to subventrally; peristome thin, slightly expanded and with weak depression on parietal side. Apertural dentitions two: parietal lamella relatively strong, high, and situated near apertural lip; palatal plica moderate, low, and situated slightly deeper inside aperture (infrapalatal plica occasionally present). Umbilicus widely opened, occupies ~½ of shell width and showing all preceding whorls.

##### Differential diagnosis.

*Clostophis proboscideus* differs from *C. sankeyi* and *C. yoga* Páll-Gergely & Hunyadi, 2022 by having more numerous and more tightly coiled whorls, lower spire, and wider umbilicus. Meanwhile, *C. sankeyi* has weaker spiral striations, and *C. yoga* possesses no apertural dentition, has a narrower umbilicus (< 1/3 of shell width), and with spiral striations throughout the protoconch (Benson 1860; Páll-Gergely and Hunyadi 2022; Preece et al. 2022).

*Clostophis proboscideus* differs from the long and descending tuba morphs of *C. multiformis* Páll-Gergely & Reischütz, 2020 and *C. laidlawi* by having a concave-conical shape, wider umbilicus, aperture opening ventrally to subventrally, spiral striations appearing at late stage of protoconch, and with two apertural dentitions (parietal and palatal). In comparison, these two latter species possess conical shells with straight sides, apertures opened sublaterally, narrow umbilicus, and with spiral striations throughout the protoconch. In addition, *C. multiformis* has only a parietal lamella, while *C. laidlawi* possesses five apertural dentitions (parietal, angular, upper palatal, lower palatal, and columellar) (Collinge 1902; van Benthem Jutting 1961; Páll-Gergely et al. 2020).

##### Distribution.

This species is currently known from several limestone outcrops in central Thailand.

##### Remarks.

The correct publication date of *C. proboscideus* has been specified in Jirapatrasilp et al. (2023: 24). This species was described based on three specimens: the holotype and two paratypes. The paratypes are photographed herein, one with a broken palatal wall and the other still intact (Fig. 3A–C). However, when we revisited the type locality, we found only two empty shells (Fig. 4A–C).

Shell variations were observed from specimens from Khao Patawi, Uthai Thani Province. These specimens possessed a general shell form similar to the type specimens but tended to have wider spaces between radial striations on the last whorl (~14–20), and the infrapalatal plica may be present. When an infrapalatal is present, it is located close to a larger plica, and the infrapalatal varies from a noticeable but low ridge (Fig. 5A) to weak and inconspicuous (Fig. 5B, C) or without an infrapalatal plica (Fig. 5D). These variations occur syntopically, and we consider this as intrapopulation variation.

#### ﻿ Clostophis rhynchotes

Taxon classificationAnimaliaStylommatophoraHypselostomatidae

Tongkerd & Panha sp. nov.

FC546542-DCD6-53CB-948C-EC093483CA20

https://zoobank.org/B7590776-7C97-4372-AC9E-CE1B98BCBC04

Figs 6, 7, Table 3

##### Type material.

**Figure 6.**
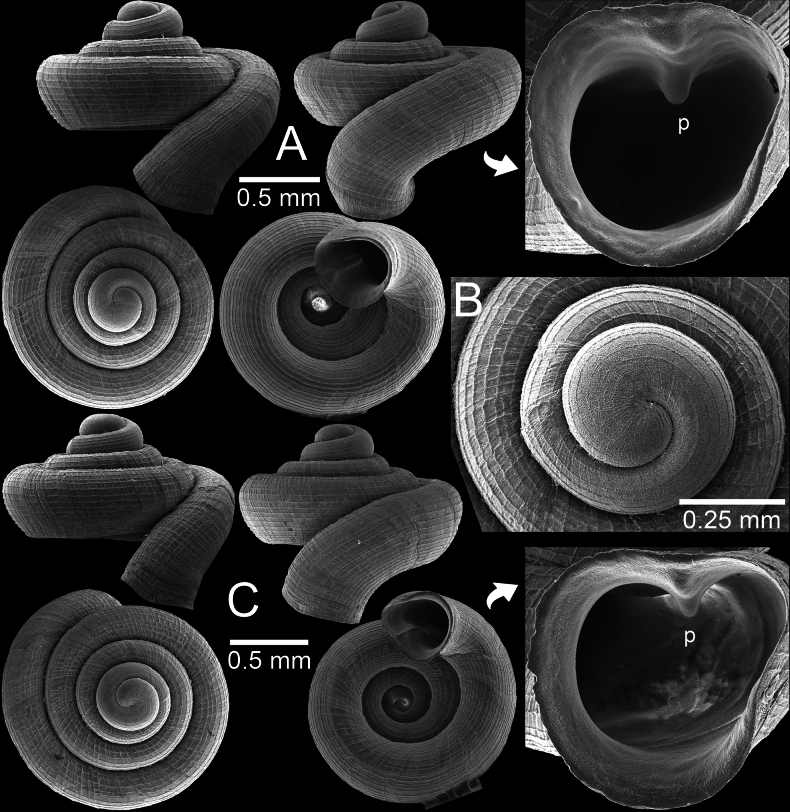
*Clostophis rhynchotes* sp. nov. from Nakhon Sawan Province. A, B. Holotype CUMZ 14460 with enlarged aperture: B. Protoconch and earlier whorl sculptures; C. Paratype CUMZ 14459 from the type locality.

**Figure 7.**
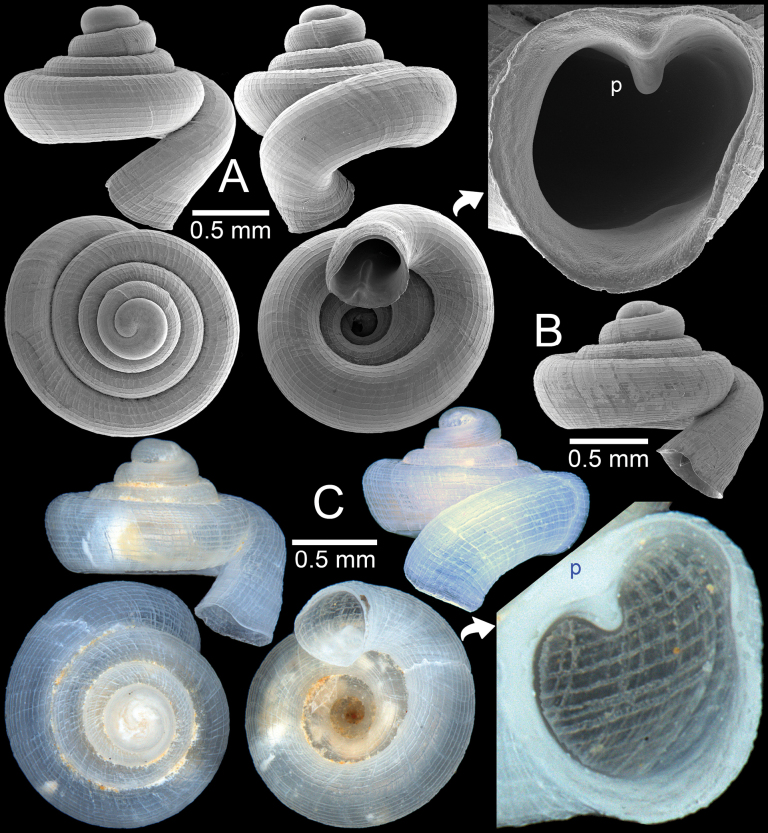
*Clostophis rhynchotes* sp. nov. from Nakhon Sawan Province. A. Paratype CUMZ 14437 with enlarged aperture; B. Paratype CUMZ 14436 from the type locality; C. Paratype CUMZ 14464 under light microscope showing colourless and translucent shell and with enlarged aperture.

***Holotype*.** Thailand • height 1.3 mm (including tuba), width 1.5 mm (Fig. 6A, B); Wat Khao Chakkachan Wanaram, Chum Ta Bong District, Nakhon Sawan Province; 15°35'44.4"N, 99°32'39.3"E; P. Tongkerd leg.; CUMZ 14460. ***Paratypes*.** Thailand • 1 shell (Fig. 6C); same data as for holotype; CUMZ 14459. • 1 shell (Fig. 7B); same data as for holotype; CUMZ 14436. • 1 shell (Fig. 7A); same data as for holotype; CUMZ 14437. • 29 specimens in ethanol (Fig. 7C; COI accession no. PV698339); same data as for holotype; CUMZ 14464 (COI accession number PV698339). • 2 shells; same data as for holotype; NHMUK 20250359. • 2 shells; same data as for holotype; SMF.

##### Diagnosis.

Shell depressed concave-conical, penultimate whorl slightly sunken into last whorl, long and descending tuba, 12–14 spiral striations, peristome weakly expanded, with only parietal lamella, and wide umbilicus.

##### Description.

Shell depressed, concave-conical, colourless; spire growing regularly and last whorl broadly expanded. Shell height 1.3–1.4 mm (including tuba) and shell width 1.4–1.5 mm. Apex large and rounded; protoconch ~2 whorls, pitted and sculptured with prominent spiral striae. Whorls ~4–4¾ (excluding tuba) weakly shouldered and convex; suture wide and deep. Shell surface with strong, elevated, continuous, equidistant spiral striae (12–14 on body whorl in frontal view), and crossed with weaker and irregular radial growth lines. Growth lines on earlier whorls rather strong, making reticulated surface. Penultimate whorl slightly sunken into last whorl. Last whorl with slight indication of blunt shoulder; tuba short, < ¼ whorl, strongly descending and twisted. Aperture subovate, open ventrally to subventrally; peristome thin and slightly expanded on columellar side and with weak depression on parietal side. Apertural dentition one: parietal lamella strong and tall with low ridge near peristome edge then gradually taller deep inside aperture. Umbilicus widely opened, occupies ~½ of shell width and showing all preceding whorls.

##### Differential diagnosis.

*Clostophis rhynchotes* sp. nov. differs from *C. proboscideus*, *C. sankeyi*, and *C. yoga* by having a depressed conical spire, strong parietal lamella, and 12–14 spiral striae on last whorl. In comparison, these other three species possess conical spires with 18–20 spiral striae on last whorl. In addition, *C. proboscideus* has a parietal lamella and a strong palatal plica, while *C. sankeyi* and *C. yoga* generally have no dentition, but parietal lamella and palatal plica may be weakly present in *C. sankeyi* (Páll-Gergely et al. 2020).

*Clostophis rhynchotes* sp. nov. also differs from *C. udayaditinus* Sutcharit & Panha, 2025 from Cambodia by having slightly concave-sided shell, penultimate whorl slightly sunken into last whorl, 12–14 spiral striae on last whorl, and only one parietal lamella, while *C. udayaditinus* has a strongly concave shell, the penultimate whorl sunken into last whorl, 18–24 spiral striae on last whorl, and four dentitions (hooked parietal, infraparietal, palatal, and hooked columellar) (Sutcharit et al. 2025).

##### Distribution.

This new species is currently known only from the type locality. There is a small limestone outcrop (700 m long and 250 m wide), and a hill covered with low vegetation, surrounded by a housing area and temple, and with low disturbance.

##### Etymology.

The specific name *rhynchotes* is from the Greek word *rhynchos* meaning ‘snout’ and the suffix –*otes*; it refers to the tuba of the last whorl, which resembles the snout of a tapir.

##### Remarks.

Although *C. rhynchotes* sp. nov. shares a depressed shell, a long descending tuba, and spirally striated protoconch with *A. rhamphodontis* sp. nov., it is distinguished from the latter by the prominent spiral striae crossed by less prominent growth lines without forming a rectangular pattern, and with very few dentitions. These characteristics indicate the placement of this new species within the genus *Clostophis*.

#### ﻿ Acinolaemus

Taxon classificationAnimaliaStylommatophoraHypselostomatidae

Thompson & Upatham, 1997

19803BBA-1E93-52D1-AC6D-8935AF7197A3

Acinolaemus Thompson & Upatham, 1997: 223, 224. Schileyko 1998: 255. Panha and Burch 2005: 39. Vermeulen et al. 2007: 86.

##### Type species.

*Acinolaemus ptychochilus* Thompson & Upatham, 1997, by original designation.

##### Remarks.

The genus currently contains 11 species (MolluscaBase 2025) with few to many apertural dentitions, a shell with ascending to descending tuba or without tuba, and a shell surface with rectangular reticulations to prominent spiral striations (Table 4; Thompson and Upatham 1997). These variations are possibly the cause that rendered the generic boundary ambiguous and necessitates intensive systematic revision.

**Table 4.** Species list, diagnostic characters, and type localities of *Acinolaemus* species. Numbers in species column refer to those in Fig. 2B. The superscript numbers indicate references: 1 = Thompson and Upatham (1997); 2 = Schileyko (1998); 3 = Vermeulen et al. (2007); 4 = Vermeulen et al. (2019); 5 = Changlom et al. (2019); 6 = Tongkerd et al. (2024); 7 = this study.

Species	Shell shape	Shell sculpture	Number of dentitions on			Palatal tubercle	Type locality
			parietal wall	palatal wall	columellar wall		
1. *A. cryptidentatus*Changlom et al., 2019^5,6,7^	conical, slightly concave side, no tuba	rectangular reticulation	3 (parietal, angular, infraparietal)	4 (3 palatals, basal)	1 columella	present	Thailand, Mae Hong Son
2. *A. dayanum* (Stoliczka, 1871)^6^	concave-conical, no tuba	rectangular reticulation	3 (parietal, angular, infraparietal)	4 (3 palatals, basal)	1 columella	present	Myanmar, Mon
3. *A. mueangonensis*Changlom et al., 2019^5,7^	conical, straight to concave side, no tuba	rectangular reticulation	3 (parietal, angular, infraparietal)	4 (3 palatals, basal)	1 or 2 columellae	present	Thailand, Chiang Mai
4. *A. ptychochilus* Thompson & Upatham, 1997^1,2,7^	conical, slightly concave side, no tuba	rectangular reticulation	3 (parietal, angular, infraparietal)	5 (4 palatals, basal)	3 (columellae)	present	Thailand, Chiang Mai
5. *A. corusticorus*, sp. nov.^7^	conical, slightly concave side, no tuba	rectangular reticulation	3 (parietal, angular, infraparietal)	3 or 4 palatals	2 (columellae)	present	Thailand, Tak
6. *A. rhamphodontis* sp. nov.^7^	depressed conical, concave side, long and descending tuba	rectangular reticulation	3 (parietal, angular, infraparietal)	3 (2 palatals, basal)	2 (columellae)	present	Thailand, Tak
7. *A. carcharodon*Vermeulen et al., 2007^4^	depressed conical, curved side, short descending tuba	prominent spiral striations	2 (parietal, angular)	2 (parietal, basal)	absent	absent	Vietnam, Kien Giang
8. *A. pyramidalis* (Vermeulen et al., 2007)^3^	conical, slightly concave side, short and descending tuba	prominent spiral striations	2 (parietal, angular)	2 palatals	1 columella or absent	absent	Vietnam, Kien Giang
9. *A. rectus*Vermeulen et al., 2019^3^	conical, slightly concave side, short and ascending tuba	prominent spiral striations	2 (parietal, angular)	2 palatals	1 columella	absent	Cambodia, Kampot
10. *A. rhamphodon* Thompson & Upatham, 1997^1^	concave-conical, no tuba	prominent spiral striations	2 (parietal, angular)	2 palatals	1 columella	weak	Thailand, Chachoengsao
11. *A. stenopus* Thompson & Upatham, 1997^1^	conical, slightly concave side, no tuba	prominent spiral striations	2 (parietal, angular)	2 palatals	1 columella	present	Thailand, Chanthaburi
12. *A. colpodon* Thompson & Upatham, 1997^1^	conical, slightly concave side, long and ascending tuba	prominent spiral striations	1 angular	1 palatal	1 columella	weak	Thailand, Rayong
13. *A. sphinctinion* Thompson & Upatham, 1997^1^	conical, straight side, very short and ascending tuba	prominent spiral striations	2 (angulo-parietal, infraparietal)	2 palatals	1 columella	absent	Thailand, Pra Chuap Khiri Khan

#### ﻿ Acinolaemus ptychochilus

Taxon classificationAnimaliaStylommatophoraHypselostomatidae

Thompson & Upatham, 1997

84775BC6-930E-5846-B095-4E48015FC6FD

Figs 8, 9, Table 4

**Figure 8.**
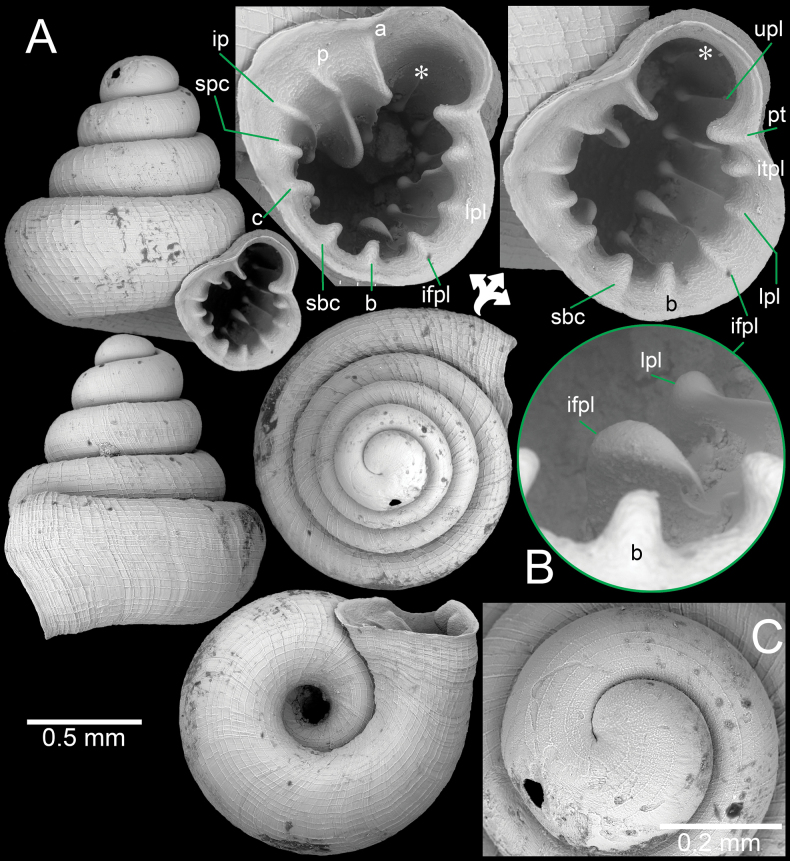
*Acinolaemus ptychochilus* from Chiang Mai Province. A. Holotype UF 113502 with enlarged aperture from three different angles; B. Inset of hooked infrapalatal plica; C. Protoconch and earlier whorl sculptures. An asterisk (*) indicates small lamella and plica situated in posterior chamber. Photo credit: B. Páll-Gergely.

**Figure 9.**
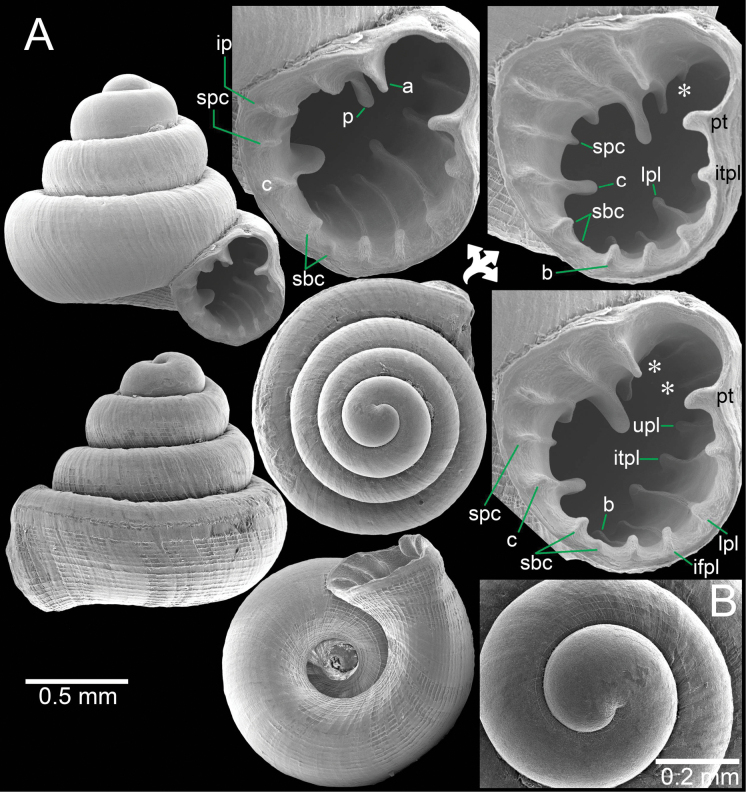
*Acinolaemus ptychochilus* from Chiang Mai Province. A. Specimen CUMZ 15360 with enlarged aperture from three different angles; B. Protoconch and earlier whorl sculptures. An asterisk (*) indicates small lamella and plica situated in posterior chamber.

**Figure 10.**
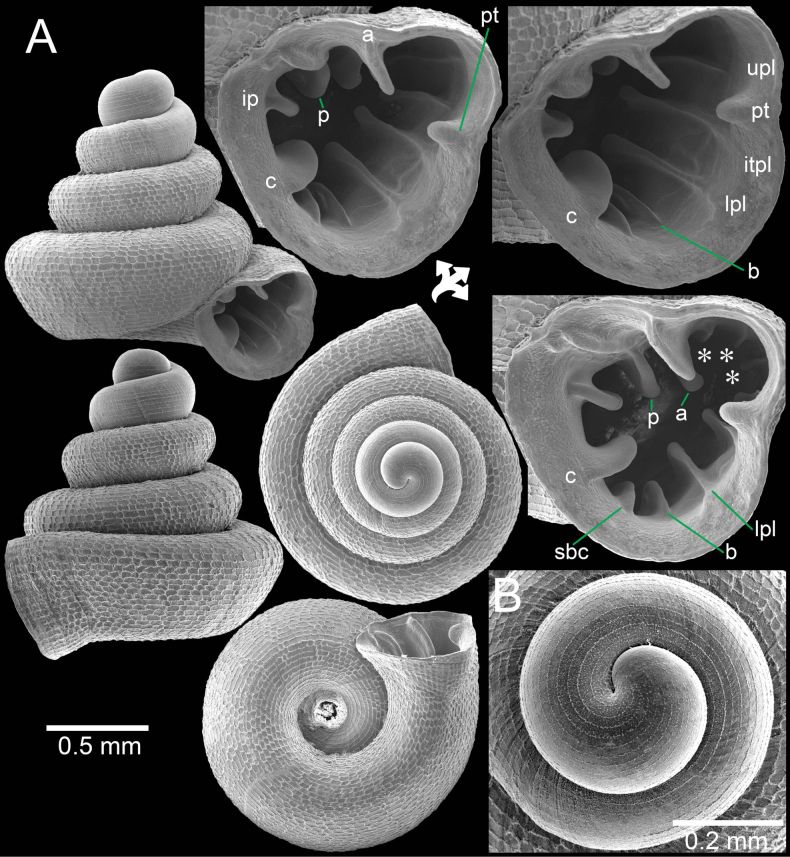
*Acinolaemus cryptidentatus* from Mae Hong Son Province. A. Specimen CUMZ 15362 with enlarged aperture from three different angles; B. Protoconch and earlier whorl sculptures. An asterisk (*) indicates small lamella and plica situated in posterior chamber.

**Figure 11.**
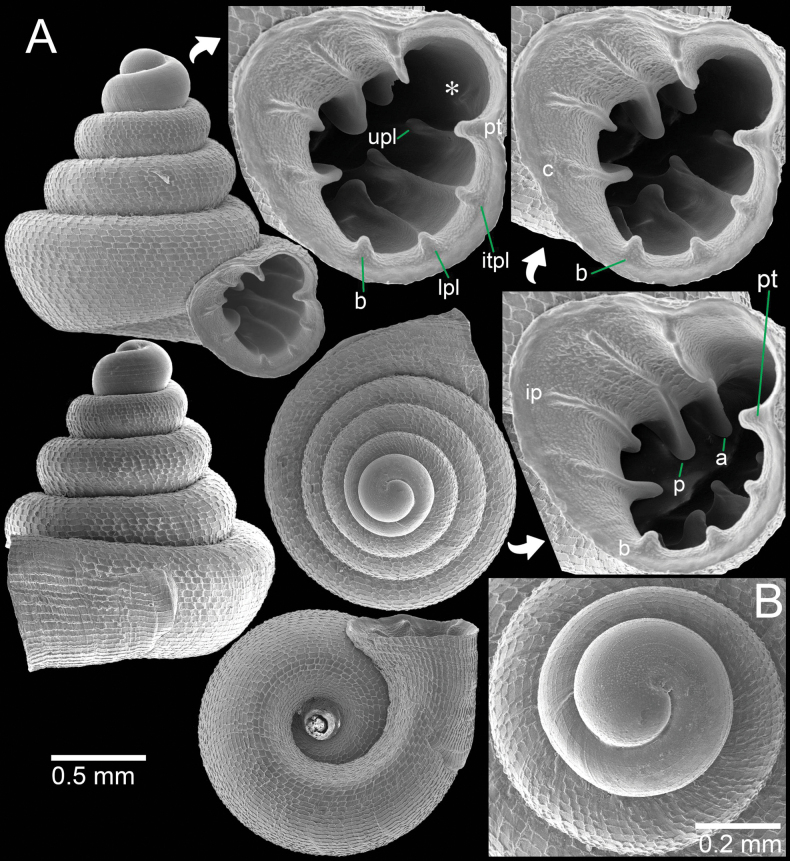
*Acinolaemus mueangonensis* from the type locality in Chiang Mai Province. A. Specimen CUMZ 15361 with enlarged aperture from three different angles; B. Protoconch and earlier whorl sculptures. An asterisk (*) indicates small lamella and plica situated in posterior chamber.

Acinolaemus ptychochilus Thompson & Upatham, 1997: 225, 226, figs 7–11. Type locality: Ban Prang Ma-O, Doi Pha San Sao (Mountain), Chiang Mae [= Chiang Mai] Province, Thailand.
Acinolaemus ptychochilus —Schileyko 1998: 255, fig. 316.

##### Type material examined.

***Holotype*.** Thailand • height 1.5 mm, width 1.3 mm (Fig. 8); Doi Pha San Sao, Ban Prang Ma-O, Chiang Mai Province; F.G. Thompson leg.; UF 113502.

##### Other material.

Thailand • 4 shells (Fig. 9); limestone near Chai Prakan Highway Division (~43 km to Chieng Dao District), Chai Prakan District, Chiang Mai Province; S. Panha leg.; CUMZ 15360.

##### Diagnosis.

Shell concave-conical, peristome weakly expanded. Apertural dentitions eleven or more, long and reaching peristome edge in form of prominent knobs: three on parietal wall, palatal tubercle, four on palatal wall, one basal, and two on columellar wall.

##### Re-description.

Shell conical, with concave sides, colourless; spire low to high and growing regularly; last whorl expanded. Shell height 1.3–1.4 mm and shell width 1.4–1.5 mm. Apex blunt; protoconch ~1½–2 whorls, with narrow and fine spiral striae. Whorls ~4–5, rounded and convex; suture wide, well impressed, and deep. Shell surface sculptured with equidistantly spaced spiral striae, crossed with irregular radial growth lines making a rectangular reticulated sculpture throughout shell. Penultimate whorl regularly coiled; last whorl rounded. Aperture subovate; peristome thickened, slightly expanded and with weak depression on parietal side. Aperture with approx. ten or eleven dentitions and with strong knobs reaching peristome edge. Parietal wall with three lamellae: parietal lamella large, outer part low, inner part long with tall ridge and located deeper inside aperture; infraparietal lamella long and low; angular lamella long, consisting of two peaks of prominent tall ridges which are interrupted by a low wide ridge. Palatal tubercle rectangular, situated at peristome edge and continuous with upper palatal plica. Three tiny low plicae present (not reaching peristome edge) in sinulus. Palatal wall with four plicae: upper-, inter-, lower-, and infra-palatal plicae connecting peristome edge with prominent tubercles, then continuing with narrow, low ridge, and becoming a stronger fold deep inside aperture. Folding of inter- and lower-palatal plicae larger than upper- and infra-palatal plicae. Infrapalatal plica hooked (in holotype). Basal plica a low ridge and similar to infrapalatal plica. Columellar wall with three or four lamellae: columellar lamella strong, distinct, and tall ridge; supracolumellar and one or two subcolumellar lamellae present as low ridges. Umbilicus widely perforated, ~⅓ of shell width, rounded and deep.

##### Distribution.

*Acinolaemus ptychochilus* is known from the type locality in Chiang Dao District, Chiang Mai Province in northern Thailand (Thompson and Upatham 1997).

##### Remarks.

The specimens from Chai Prakan District, which is ca 30 km north of the type locality, have only faint protoconch and teleoconch sculptures due to shell weathering. However, the original sculpture remains near the suture and around the umbilical area. These specimens differ slightly from the holotype (Fig. 8A) in having a slightly more depressed shell, a less expanded last whorl, infrapalatal plica that is not hooked (hooked in the type), and two subcolumellar lamellae (one in the type); however, the remaining apertural dentitions are identical to those in the holotype.

#### ﻿ Acinolaemus cryptidentatus

Taxon classificationAnimaliaStylommatophoraHypselostomatidae

Changlom, Chan-ard & Dumrongrojwattana, 2019

9ACF3662-3683-5345-BF24-8E1F6266AFEE

Fig. 10, Table 4

Acinolaemus cryptidentatus Changlom, Chan-ard & Dumrongrojwattana, 2019: 158, 159, fig. 2. Type locality: Tham Wua (Wua Cave), Mueang District, Mae Hong Son Province, Thailand. Tongkerd et al. 2024: 164, figs 1b, 2b.

##### Material examined.

Thailand • 3 shells (Fig. 10A, B); Mae La Na Cave, Pang Mapha District, Mae Hong Son Province; 19°34'30.8"N, 98°12'56.3"E; S. Panha leg.; CUMZ 15362.

##### Diagnosis.

Shell concave-conical, peristome weakly expanded, rectangular reticulated sculpture present on shell surface. Apertural dentitions nine: three on parietal wall, three on palatal wall, one basal, and two on columellar wall. Palatal tubercle prominent and located between upper- and inter-palatal plicae.

##### Re-description.

Shell weakly concave-conical, colourless; spire high and growing regularly; last whorl expanded. Shell height 1.6–1.8 mm and shell width 1.5–1.6 mm. Apex blunt; protoconch ~2 whorls with conspicuous spiral striae. Whorls ~4–5, bluntly shouldered, and convex; suture wide, well impressed, and deep. Shell surface sculptured with equidistantly spaced spiral striae, crossed with discontinuous and irregular radial ridge-like growth lines making rectangular reticulated sculpture throughout shell. Penultimate whorl regularly coiled to slightly sunken into last whorl. Last whorl rounded to weakly shouldered and flattened below periphery. Aperture subovate; peristome weakly expanded. Aperture with eight dentitions. Parietal wall with three lamellae: parietal lamella large, outer part low and reaching peristome edge, inner part strongly developed with long tall ridge located deeper inside aperture; infraparietal lamella prominent with a high ridge; angular lamella slightly curved, reaching peristome edge, a strong, tall ridge, long deeper inside aperture and with deep incision medially. Palatal tubercle strong, triangular, and situated on peristome edge between upper- and inter-palatal plicae. Three tiny and low plicae present in sinulus. Palatal wall with three plicae: upper-, inter-, and lower-palatal plicae with tall ridges and situated slightly inside aperture. Lower palatal plica tall and more prominent than upper- and inter-palatal plicae. Basal plica prominent with tall ridge. Columellar wall with two lamellae: columellar lamella is a very tall and distinct ridge; subcolumellar lamella a strong ridge and almost same size as basal plica. Umbilicus widely perforated, ~⅓ of shell width, rounded and deep.

##### Distribution.

*Acinolaemus cryptidentatus* is known from the type locality at Tham Wua Cave, Mae Hong Son Province in northern Thailand (Changlom et al. 2019), and subsequently reported from Shan State, Myanmar (Tongkerd et al. 2024).

##### Remarks.

The specimens from Mae La Na Cave in Pang Mapha District, which is ca 15 km east of the type locality are almost identical to the type specimen in shell shape, sculpture, and most of the apertural dentitions. However, these specimens have a prominent subcolumellar lamella, which was probably overlooked in the original description.

#### ﻿ Acinolaemus mueangonensis

Taxon classificationAnimaliaStylommatophoraHypselostomatidae

Changlom, Chan-ard & Dumrongrojwattana, 2019

6482881F-8AD2-5706-BCF8-AA1AB433E805

**Figure 12.**
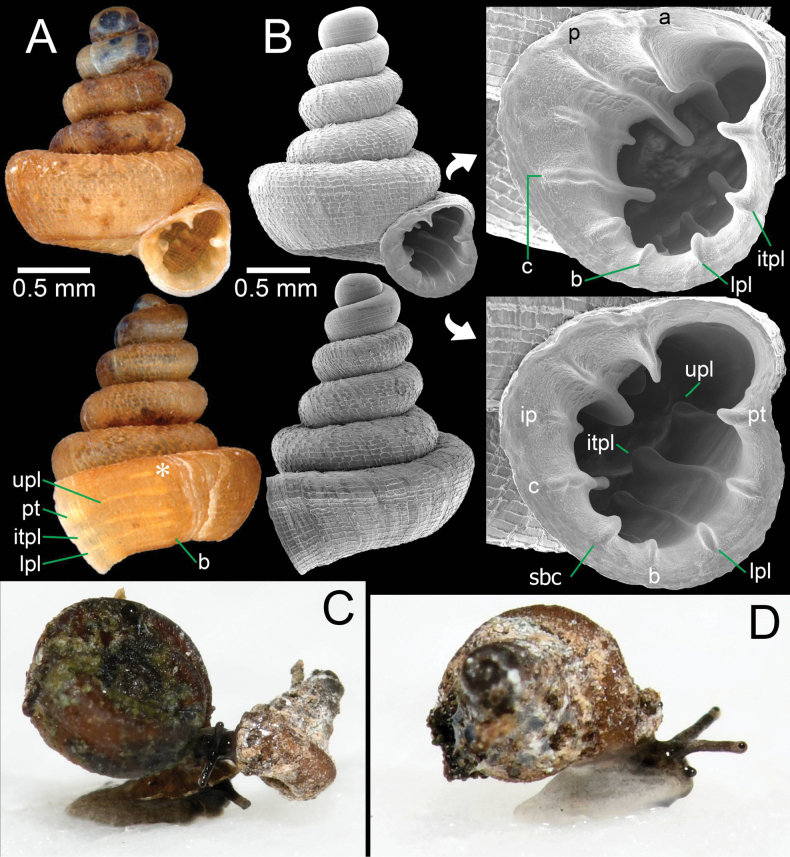
*Acinolaemus mueangonensis* from Tak Province. A. Specimen CUMZ 14455.1 under light microscope showing brownish shell and plicae; B. Specimen CUMZ 14455.3 with enlarged aperture from two different angles; C, D. Living snails CUMZ 14455.4: C. Snail crawling on *Hypselostoma pendulum*; D. Crawling on wet paper towel (shell width ~1.8 mm). An asterisk (*) indicates small lamella and plica situated in posterior chamber.

**Figure 13.**
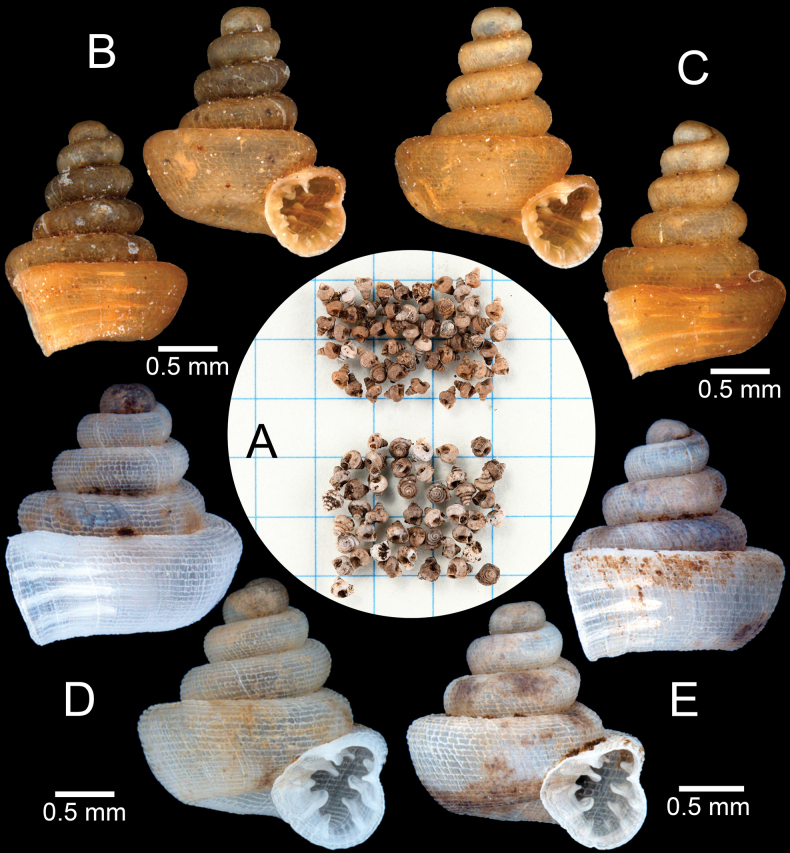
A. Synoptic photo of sympatric *Acinolaemus* species, specimens sorted from soil sample collected at Tak Province; B, C. *Acinolaemus mueangonensis*, specimen CUMZ 14455.1 with brownish shell in lateral and apertural views showing long plicae visible throughout the translucent shell; D, E. *Acinolaemus corusticorus* sp. nov., paratype CUMZ 14455.2 with colourless shell in lateral and apertural views showing short plicae visible throughout the translucent shell.

Figs 11, 12, 13B, C, Table 4

Acinolaemus mueangonensis Changlom, Chan-ard & Dumrongrojwattana, 2019: 159–161, fig. 3. Type locality: Tham Mueang On [Mueang On Cave], Mae On District, Chiang Mai Province, Thailand.
Acinolaemus muangonensis [sic]—Changlom et al. 2019: 155 (abstract), 160 (figure caption).

##### Material examined.

Thailand • 8 shells (Fig. 11A, B); Mueang On Cave, Ban Sa Ha Khon, Mae On District, Chiang Mai Province; 18°47'13.2"N, 99°14'16.6"E; S. Panha leg.; CUMZ 15361 [type locality]. • 53 shells (Figs 12A, 13B, C); Phra Phutthabat Doi Khao Nam, Ban Na Subdistrict, Sam Ngao District, Tak Province; 17°14'54.7"N, 98°56'17.1"E; P. Tongkerd leg.; CUMZ 14455.1. • 1 shell (Fig. 12B); same data as preceding; CUMZ 14455.3. • 10 specimens in ethanol (Fig. 12C, D); same data as preceding; CUMZ 14455.4.

##### Diagnosis.

Shell concave-conical, brownish and with rectangular reticulated sculpture. Apertural dentitions nine, long and reaching peristome edge: three on parietal wall, three on palatal wall, one basal and one on columella wall. Palatal tubercle continuous with upper palatal plica.

##### Re-description.

Shell conical, concave-sided, brownish to pale brown; spire low to high, growing regularly, and sometimes slightly bent; last whorl expanded. Shell height 1.8–1.9 mm and shell width 1.7–1.8 mm. Apex blunt; protoconch spirally striated, ~2. Whorls ~5–6, rounded and convex; suture wide and well impressed. Shell surface sculptured with equidistantly spaced spiral striae, crossed with discontinuous and irregular radial growth lines making rectangular reticulated sculpture throughout shell. Penultimate whorl regularly coiled or sunken into last whorl. Last whorl bluntly shouldered and flattened below periphery. Aperture subcircular; peristome thickened and slightly expanded. Aperture with eight dentitions and with strong knobs reaching peristome edge. Parietal wall with three lamellae: parietal lamella large, outer part low, and inner part strongly developed and high, long deeper inside aperture; infraparietal lamella long and low; angular lamella prominent, high, long deeper inside aperture, and with slightly narrow and low ridge in middle. Palatal tubercle triangularly shaped, situated on peristome edge and connected to upper palatal plica. One or two tiny and low plicae (not reaching peristome edge) may be present in sinulus. Palatal wall with three plicae: upper-, inter-, and lower-palatal plicae ending on peristome edge as prominent tubercles, then continuing as narrow and low, but becoming strong deeper inside aperture. Basal plica low and long deeper inside aperture. Columellar lamella strong, high, and continuing deep inside aperture. Umbilicus widely perforate, ~⅓ of shell width, rounded and deep.

##### Living animal.

Snails are typically stylommatophoran with two pairs of tentacles. Upper tentacles are long, stout, cylindrical tubes, dark greyish, and with dark eye spots on the tip (Fig. 12C, D). Lower pairs are very short to knob-shaped and can be seen clearly in full extension in moving snails. Animal with short body, anterior-dorsal side with grey to blackish pigmentation, and posterior body and foot pale greyish to semi-translucent. The snails tend to cover their shell with soil, mud, or dirt.

##### Distribution.

*Acinolaemus mueangonensis* has a distribution beyond its type locality in northern Thailand (Changlom et al. 2019), since during this study several specimens were collected in soil samples taken at the base of limestone cliffs and outcrops in Tak Province, ca 170 km south of the type locality.

##### Remarks.

The specimens from the type locality examined herein have shell sculpture and apertural dentition similar to the original description and the type specimens (Changlom et al. 2019), except for the presence of a tiny plica in the sinulus, which was not mentioned in the original description.

The specimens from central Thailand (Tak population) show the same distinguishing characters as the type specimens: a brownish shell, rectangular reticulated sculpture, and long denticles deep inside the aperture and becoming strong knobs when reaching the peristome edge. In addition, the parietal lamella has a low ridge near the peristome that becomes a tall ridge inside the aperture, and the angular lamella has a high ridge with a narrow and low ridged incision in the middle. However, the central Thailand population (Fig. 12A, B) differed from typical specimens (Fig. 11A, B) in having a more concave-sided shell, a more elevated and slender spire (height 2.1–2.2 mm and width 1.7–1.8 mm), and the penultimate whorl slightly sunken into the last whorl. Additionally, the central Thailand population has a basal plica instead of a subcolumellar lamella, while a typical shell has a subcolumellar lamella instead of a basal plica. However, the distinction between the subcolumellar lamella and the basal plica is sometimes difficult and not a reliable character for species distinction and is subject to intraspecific variability. Therefore, we provisionally recognise the central population as conspecific with *A. mueangonensis*, since no concrete difference in morphology could be observed. DNA sequence data are needed to resolve this ambiguity.

#### ﻿ Acinolaemus corusticorus

Taxon classificationAnimaliaStylommatophoraHypselostomatidae

Tongkerd & Panha sp. nov.

B875F10A-A735-5F09-93EA-1DD2F53525CA

https://zoobank.org/78BD0129-3EAB-4593-AA77-8FCED5DCD0DA

**Figure 14.**
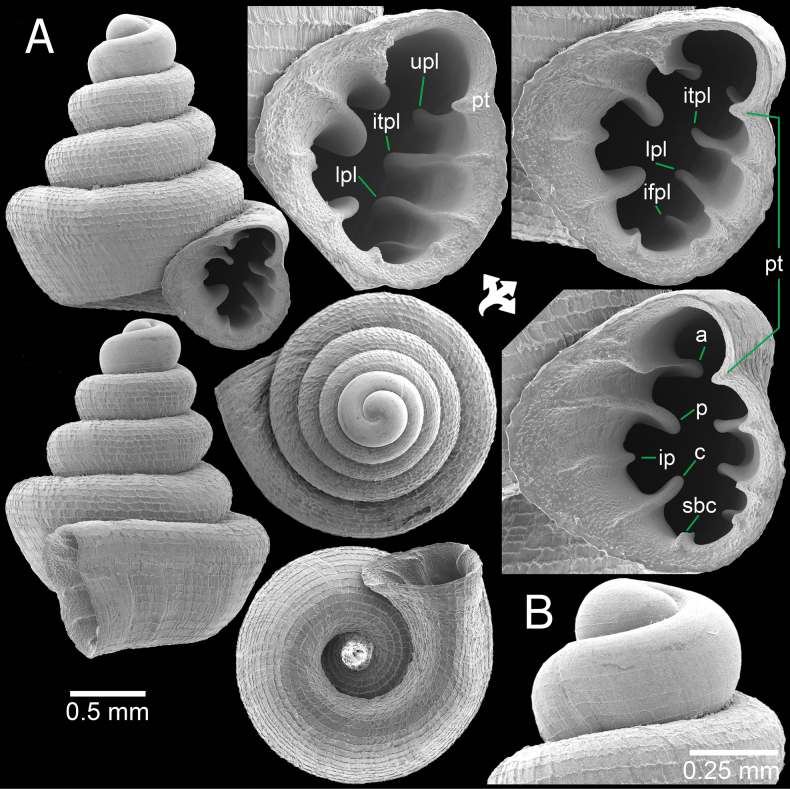
*Acinolaemus corusticorus* sp. nov. from Tak Province. A, B. Holotype CUMZ 15363.1 with enlarged aperture from three different angles and B. Protoconch and earlier whorl sculptures.

**Figure 15.**
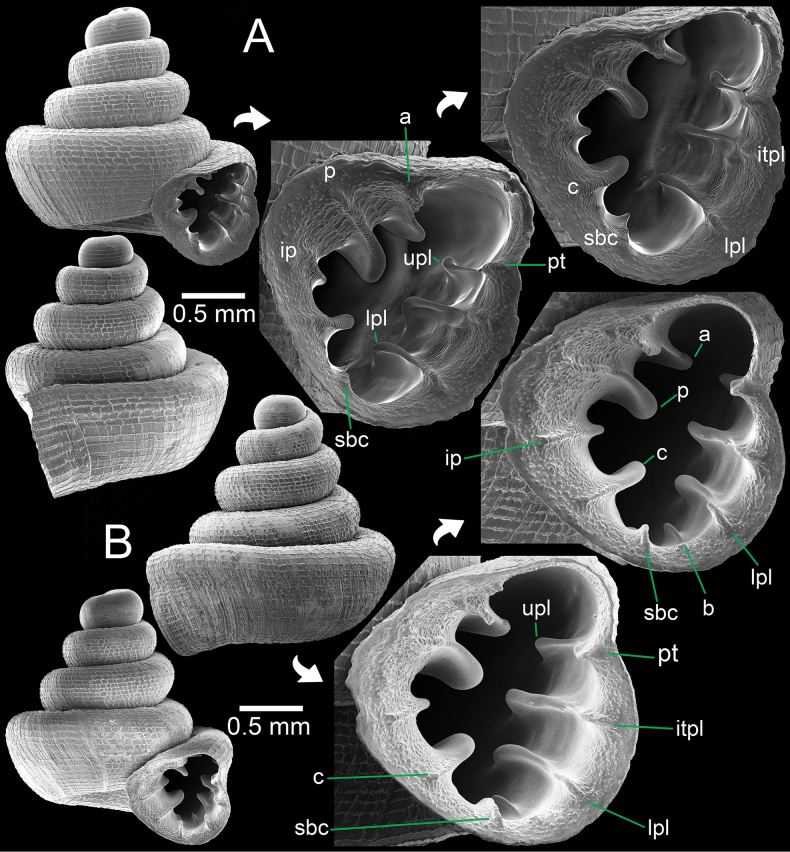
*Acinolaemus corusticorus* sp. nov. from the type locality. A. Paratype CUMZ 15363.2 with enlarged aperture from two different angles (without basal plica); B. Paratype CUMZ 15363.2 with enlarged aperture from two different angles (with basal plica).

Figs 13D, E, 14, 15, Table 4

##### Type material.

***Holotype*.** Thailand • height 2.2 mm, width 1.9 mm (Fig. 14A, B); Phra Phutthabat Doi Khao Nam, Ban Na, Sam Ngao District, Tak Province; 17°14'54.7"N, 98°56'17.1"E; P. Tongkerd leg.; CUMZ 15363.1. ***Paratypes*.** Thailand • 2 shells (Fig. 15A, B); same data as for holotype; CUMZ 15363.2. • 45 shells (Fig. 13D, E); same data as for holotype; CUMZ 14455.2. • 2 shells; same data as for holotype; NHMUK 20250360. • 2 shells; same data as for holotype; SMF.

##### Diagnosis.

Shell concave-conical, colourless, and with rectangular reticulations. Nine apertural dentitions, reaching peristome edge: three on parietal wall, palatal tubercle, four on palatal wall, and two on columellar wall. Palatal tubercle continuous with upper palatal plica.

##### Description.

Shell concave-conical, colourless; spire high and growing regularly; last whorl expanded. Shell height 2.1–2.3 mm and shell width 1.8–1.9 mm. Apex blunt; protoconch ~2 whorls with fine spiral striae. Whorls 4–5, rounded and convex; suture wide and well impressed. Shell surface sculptured with equidistantly spaced spiral striae, crossed with irregular radial growth lines making a rectangular reticulated sculpture throughout shell. Penultimate whorl regularly coiled; last whorl bluntly shouldered and flattened below periphery. Aperture subovate; peristome thickened and slightly expanded. Aperture with nine or ten dentitions and with more or less strong knobs reaching peristome edge. Parietal wall with three lamellae: parietal lamella large, strongly developed, and high and long deeper inside aperture; infraparietal lamella long and low; angular lamella relatively smaller than parietal lamella, long, low near peristome edge, somewhat sinuous, and higher deeper inside aperture. Palatal tubercle strongly developed with triangular shape and continuous with upper palatal plica. Palatal wall with four plicae: upper-, inter-, lower-, and infra-palatal plicae connecting with peristome in the form of prominent tubercles then continuing as narrow and low, becoming strong and high inside aperture. Inter- and lower-palatal plicae much larger than upper palatal plica, infrapalatal plica smallest or may be absent (Fig. 15A). Basal plica may be present with small and low ridge. Columellar wall with two lamellae: columellar lamella strong and distinct, continuing deep inside aperture; subcolumellar lamella small, low. Umbilicus widely perforate, ~⅓ of shell width, rounded and deep.

##### Differential diagnosis.

This new species can be distinguished from *A. cryptidentatus* from northern Thailand by having major dentitions (on parietal, palatal, and columellar walls) that become strong knobs when reaching peristome edge, palatal tubercle continuous with upper palatal plica, and without tiny plicae inside the sinulus. In comparison, *A. cryptidentatus* possesses dentitions that do not reach the peristome edge, a palatal tubercle situated between upper- and inter-palatal plicae, and with two tiny plicae inside the sinulus.

*Acinolaemus corusticorus* sp. nov. is similar to *A. dayanum* (Stoliczka, 1871) from Myanmar and *A. mueangonensis* from northern Thailand in shell form and sculpture. It differs by having a colourless shell without cervical crest (a swelling or convex ridge on the last whorl behind the expanded lip); parietal lamella has a thick and high ridge; angular lamella has a continuously high ridge and without incision; palatal plicae short (<½ of last whorl length when seen from lateral view; Fig. 13D, E), palatal plicae continue from knobs on peristome edge with short, narrow, and low ridges, and then becoming high inside the aperture. For comparison, *A. dayanum* and *A. mueangonensis* have palatal plicae continuing from peristome knobs, which are long and narrow but low ridges before becoming folds inside aperture. *Acinolaemus mueangonensis* possesses a brownish and conical to elevated conical shell; parietal lamella has a low ridge near the peristome then becoming a tall ridge inside; angular lamella has a high ridge with narrow and low ridge in middle; palatal plicae long (>½ of last whorl length when seen from lateral view; Fig. 13B, C). *Acinolaemus dayanum* has a conical low spire with a cervical crest (a swelling or convex ridge on the last whorl behind the expanded lip); parietal lamella has a low ridge near peristome edge then gradually becoming a tall ridge inside; angular lamella has a high ridge near peristome edge and then gradually becoming a low ridge inside aperture.

*Acinolaemus ptychochilus* from northern Thailand also clearly differs from *A. corusticorus* sp. nov. in shape of parietal and angular lamellae, and in having four palatal plicae (upper-, inter-, hooked lower-, and infra-), a small basal plica, and three columellar lamellae; palatal tubercle situated between upper- and inter- palatal plicae. *Acinolaemus rhamphodon* Thompson & Upatham, 1997 and *A. stenopus* Thompson & Upatham, 1997 differ from *A. corusticorus* sp. nov. in having fewer dentitions, a very strong angular lamella, and a weak parietal lamella. Both species also possess two palatal plicae (upper- and lower-), and a columellar lamella. Finally, *A. rhamphodon* has a hooked columellar lamella, while *A. stenopus* has an elevated shell with a high spire, and an enlarged angular lamella and palatal plica that nearly enclose the sinulus.

##### Distribution.

*Acinolaemus corusticorus* sp. nov. is known only from the type locality. At this locality, the species is sympatric with five other hypselostomatid species: *Hypselostoma pendulum* (Panha & Burch, 2002), *H. khaowongensis* Panha, 1998, *A. mueangonensis*, *A. rhamphodontis* sp. nov., and *Krobylos takensis* Panha & Burch, 2004 (Panha 1998b; Panha et al 2004).

##### Etymology.

The specific name *corusticorus* is from two Latin words co- meaning ‘together or with’ and *rusticor* meaning ‘living in the country’, referring to the new species being sympatric with two other congeners.

##### Remarks.

Although this new species occurs sympatrically with *A. mueangonensis*, under the microscope it is evident that they are two distinct species. *Acinolaemus corusticorus* sp. nov. differs from sympatric congeners by having a colourless, short, and stout shell (Fig. 13A), while *A. mueangonensis* has a much smaller and more slender shell, and with pale brownish to brownish shell colour. In addition, these two species are clearly distinct in number and morphology of apertural dentitions.

#### ﻿ Acinolaemus rhamphodontis

Taxon classificationAnimaliaStylommatophoraHypselostomatidae

Tongkerd & Panha sp. nov.

93B43A14-CFBA-50E4-B61C-F690F5141DB2

https://zoobank.org/848D50A4-5B1B-457A-988A-0D07445ECDDC

**Figure 16.**
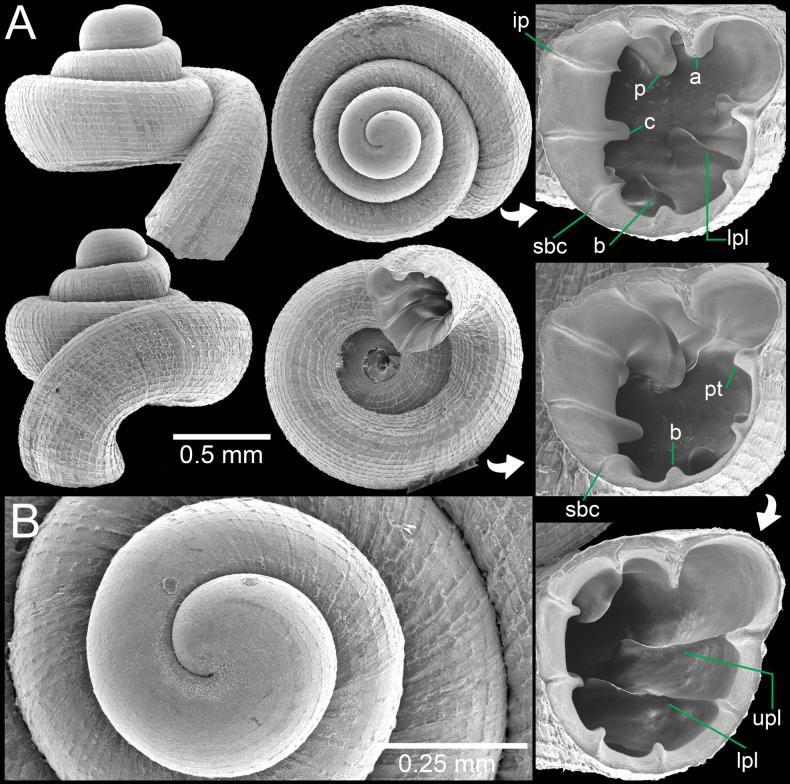
*Acinolaemus rhamphodontis* sp. nov., holotype CUMZ 14449 from Tak Province. A. Shell with enlarged aperture from two different angles; B. Protoconch and earlier whorl sculptures.

**Figure 17.**
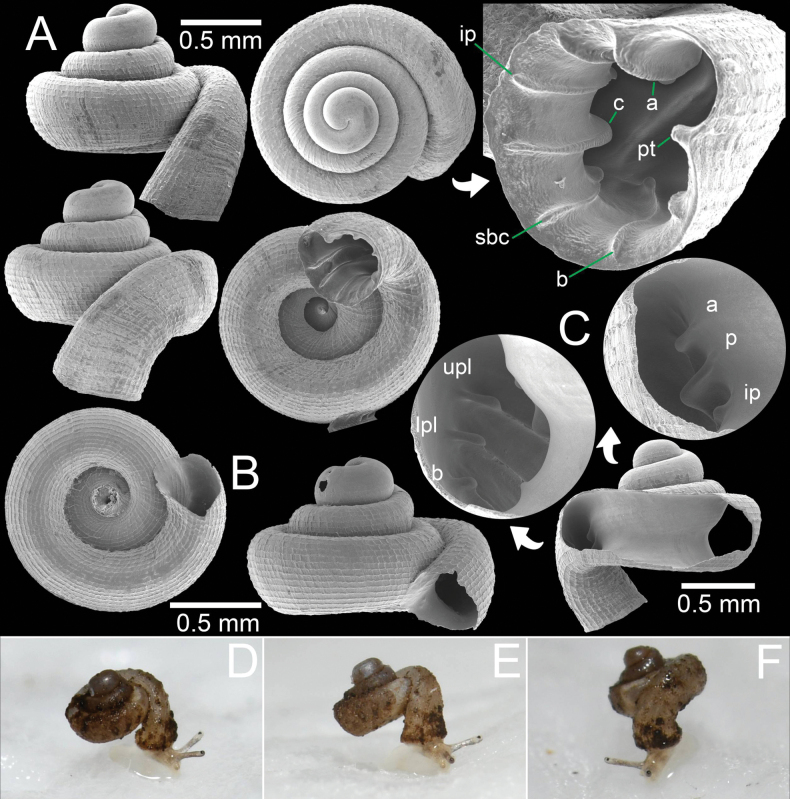
*Acinolaemus rhamphodontis* sp. nov., from Tak Province. A. Paratype CUMZ 14450 with enlarged aperture; B. Juvenile paratype CUMZ 14450 from the type locality; C. Natural broken shell with dentition on parietal and palatal walls as seen from inside the shell; D–F. Specimen CUMZ 14451 of living snails crawling on wet paper towel (different angles of same individual; shell width ~1.4 mm).

Figs 16, 17, Table 4

##### Type material examined.

***HoIotype*.** Thailand • height 1.3 mm (including tuba), width 1.4 mm (Fig. 16A, B); Phra Phutthabat Doi Khao Nam, Ban Na subdistrict, Sam Ngao District, Tak Province; 17°14'56.4"N, 98°56'16.3"E; Tongkerd leg.; CUMZ 14449. ***Paratypes*.** Thailand • 3 shells (Fig. 17A–C); same data as for holotype; CUMZ 14450. • 1 adult + 1 juvenile in ethanol (Fig. 17D–F); same data as for holotype; CUMZ 14451 (COI accession number PV698334–PV698335). • 37 adults + 3 juveniles (COI accession nos. PV698334, PV698335); same data as for holotype; CUMZ 14452. • 2 shells; same data as for holotype; NHMUK 20250361. • 2 shells; same data as for holotype; SMF.

##### Diagnosis.

Shell concave-conical, with long and descending tuba, peristome expanded. Apertural dentitions eight, all longer inside aperture: infra parietal, parietal, angular, upper- and lower-palatal, palatal tubercle, basal, subcolumellar lamella, and columellar lamella. Umbilicus wide.

##### Description.

Shell concave-conical, colourless; spire depressed and growing regularly; last whorl broadly expanded. Shell height 1.3–1.4 mm (including tuba) and shell width 1.3–1.5 mm. Apex large and rounded; protoconch ~2 whorls and with prominent spiral striae. Whorls ~4 (excluding tuba), rounded and convex; suture wide, well impressed, and deep. Shell surface sculptured with equidistantly spaced spiral striae (14–16 on body whorl in frontal view), crossed with discontinuous narrow radial growth lines making a rectangular reticulated sculpture throughout shell. Penultimate whorl regularly coiled; last whorl rounded; tuba long, ~¼ whorl or less, strongly descending and curving. Aperture subovate, open ventrally to subventrally; peristome thin, weakly expanded, and with thin depression area on parietal side. Apertural dentitions eight and all knob-shaped when reaching peristome edge. Parietal wall with three lamellae: parietal lamella long, outer part low, and then gradually becoming strong, thickened, and high inside aperture; infraparietal lamella evenly low; angular lamella strong. Palatal tubercle prominent, triangular, situated at peristome lip, and continuous with upper palatal plica. Palatal wall with two plicae: upper- and lower-palatal plicae connecting with peristome edge in form of prominent knobs, then continuing as narrow and low deeper inside aperture, eventually becoming stronger and higher at innermost ends. Basal plica narrow, and low. Columellar wall with two lamellae: columellar lamella prominent with tall ridge; subcolumellar lamella very small and low. Umbilicus widely opened, occupying ~½ of shell width and showing all preceding whorls.

##### Living animal.

Snail typically stylommatophoran with two pairs of tentacles. Upper tentacles are long, stout, cylindrical tubes, colourless to translucent, with dark eye spots on the tip. Lower pairs are very short to knob-shaped (difficult to observe in living snails). Animal with short body, anterior-dorsal side brownish while posterior body and foot paler to translucent. The snails tend to cover their shell with soil, mud, or dirt (Fig. 17D–F).

##### Distribution.

This new species is known only from the type locality, where the empty shells and one living snail were found in the soil, leaves, and twig litter at the base of a limestone cliff. The type locality is an island located in the reservoir of Bhumibol Dam, ca 45 km northwest of Tak Town. This island is ca 400 m long and 200 m wide and aligned north-south; the dry shells and the specimen were collected from the eastern slope of the island. The hills are low, with scattered land used by temples and with some exposed limestone rocks and cliffs. The vegetation on this island is dominated by low, dry, dipterocarp forest.

##### Differential diagnosis.

The shell of *A. rhamphodontis* sp. nov. is most similar to *A. dayanum* from Myanmar, and *A. cryptidentatus*, *A. mueangonensis* and *A. ptychochilus* from Thailand. They all share a rectangular reticulated shell sculpture, many long dentitions that reach the peristome edge where they form small denticles (except in *A. cryptidentatus*) and have a palatal tubercle (Thompson and Upatham 1997; Changlom et al. 2019; Tongkerd et al. 2024). The differences are that *A. rhamphodontis* sp. nov. has a long and descending tuba and a spire sunken into the last whorl. In contrast, the other four species have no tuba, and have a conical spire with straight to curved sides.

*Acinolaemus rhamphodontis* sp. nov. differs from *A. carcharodon*Vermeulen et al., 2007 and *A. pyramidalis* (Vermeulen et al., 2007) from the Mekong Delta limestone hills in Vietnam, and *A. rectus*Vermeulen et al., 2019 from Cambodia in having a long and descending tuba, long dentitions that reach the peristome edge, and a rectangular reticulated shell surface. In comparison, these three species possess a very short tuba, prominent radial ridges, and dentitions that are short and situated inside to deep inside the aperture. Additionally, *A. carcharodon* has a sunken spire and four dentitions (parietal, hooked angular, palatal, and basal), *A. pyramidalis* has a conical spire and four to five dentitions (parietal, angular, two palatals, and a very inconspicuous columellar), and *A. rectus* has a conical spire, last whorl rounded, tuba slightly ascending and five dentitions (parietal, angular, two palatals, and columellar) (Vermeulen et al. 2007, 2019).

This new species has a long descending tuba similar to several species in the *Clostophis sankeyi* species group. It differs by having rectangular reticulated shell sculpture, with eight apertural dentitions (parietal, infra parietal, angular, two palatals, basal, and two columellar) reaching the peristome edge, and with a palatal tubercle present. Furthermore, *A. rhamphodontis* sp. nov. has 14–16 dash-like spiral striae on the last whorl and the penultimate whorl not sunken, whereas *C. sankeyi*, *C. proboscideus*, *C. yoga*, and *C. udayaditinus* have 18–20 or more continuous spiral striae on the last whorl, and the penultimate whorl sunken into the last whorl. *Clostophis yoga* also has a narrower umbilicus, <1/3 of the shell width, than the new species (Benson 1860; Páll-Gergely et al. 2020; Páll-Gergely and Hunyadi 2022; Sutcharit et al. 2025). Finally, *A. rhamphodontis* sp. nov. also differs from *C. rhynchotes* sp. nov. by having a rectangular reticulated shell surface and dash-like spiral striae on the last whorl, while *C. rhynchotes* sp. nov. has a weak reticulated shell surface and continuous spiral striae on the last whorl.

##### Etymology.

The specific name *rhamphodontis* is from two Greek words: *rhamphos* meaning ‘curving beak’ and *odontos* meaning ‘tooth’. Together they refer to the tuba that curves downward and the many dentitions in the aperture.

##### Remarks.

The presence of a rectangular reticulated shell sculpture and the many dentitions reaching the peristome edge where they form small denticles and extend inside the aperture, clearly position *A. rhamphodontis* sp. nov. within the genus *Acinolaemus* (Thompson and Upatham 1997).

## ﻿Discussion

The report of these three new species from central Thailand raises some interesting questions regarding the generic boundaries and relationships between *Clostophis* and *Acinolaemus*. In the original description, *Acinolaemus* was characterised by an auriculate-shaped aperture, 1–3 dentitions on the parietal wall, an enlarged and conspicuous angular lamella, a well-developed sinulus (formed by angular lamella and palatal plica), and a spirally striated protoconch (Table 4; Thompson and Upatham 1997; Schileyko 1998). However, these diagnostic characters are not consistently expressed across all species assigned to the genus, casting doubt on their reliability for the diagnosis of the genus. For example, six species, i.e., *A. cryptidentatus*, *A. dayanum*, *A. mueangonensis*, *A. ptychochilus* (type species), *A. rhamphodontis* sp. nov., and *A. corusticorus* sp. nov. exhibit many dentitions that reach the peristome edge and extend deeper inside the aperture as long dentitions, a well-developed palatal tubercle, and with distinct rectangular reticulated shell sculptures (Thompson and Upatham 1997; Changlom et al. 2019; Tongkerd et al. 2024). In the present study, *A. mueangonensis* and *A. rhamphodontis* sp. nov. appeared as well-supported sister taxa, while *A. sphinctinion* Thompson & Upatham, 1997, *A. colpodon* Thompson & Upatham, 1997, *A. stenopus*, and *A. rhamphodon* possess only a few apertural dentitions and a palatal wall with one or two lamellae. Our phylogeny indicates that *A. rhamphodon* and *A. colpodon* form a clade with *C. rhynchotes* sp. nov. and *C. udayaditinus*, with support from the ML analysis. Interestingly, these four species share certain morphological characters such as prominent spiral striations relative to the growth lines and few apertural dentitions, which are the distinguishing characters of the *Clostophis* (Páll-Gergely et al. 2020). Moreover, three other species, namely *A. carcharodon*, *A. pyramidalis* and *A. rectus*, possess a short to long tuba, four or five apertural dentitions, and prominent spiral striations (Vermeulen et al. 2007, 2019) and these conchological characters suggest a close relationship between *Acinolaemus* and *Clostophis*, with this latter genus being defined by a colourless shell, short to long tuba, prominent spiral striations, and typically with one or more apertural dentitions (Páll-Gergely et al. 2020). Previous DNA studies by Tongkerd et al. (2004) suggest that the shell surface sculpture provides a more informative and reliable phylogenetic signal than shell shape or dentition, and this was taken into consideration in the Gojšina et al. (2025) revision. However, due to the limited number of genera and species examined in the Hypselostomatidae, it remains premature to expand further on the relationship between *Clostophis* and *Acinolaemus*.

## Supplementary Material

XML Treatment for Clostophis

XML Treatment for Clostophis proboscideus

XML Treatment for Clostophis rhynchotes

XML Treatment for Acinolaemus

XML Treatment for Acinolaemus ptychochilus

XML Treatment for Acinolaemus cryptidentatus

XML Treatment for Acinolaemus mueangonensis

XML Treatment for Acinolaemus corusticorus

XML Treatment for Acinolaemus rhamphodontis

## ﻿Additional information

### Use of AI

No use of AI was reported.

### Author ORCIDs

Piyoros Tongkerd https://orcid.org/0000-0001-9221-9293

Teerangkul Janjai https://orcid.org/0009-0007-1524-9668

Arthit Pholyotha https://orcid.org/0000-0001-6677-1164

Vukašin Gojšina https://orcid.org/0000-0002-0413-9304

Somsak Panha https://orcid.org/0000-0002-4431-2458

Chirasak Sutcharit https://orcid.org/0000-0001-7670-9540

### Data availability

All of the data that support the findings of this study are available in the main text.
